# Cheminformatics Analysis and Modeling with MacrolactoneDB

**DOI:** 10.1038/s41598-020-63192-4

**Published:** 2020-04-14

**Authors:** Phyo Phyo Kyaw Zin, Gavin J. Williams, Sean Ekins

**Affiliations:** 1 Department of Chemistry, North Carolina State University, Raleigh, NC USA; 2 Bioinformatics Research Center, North Carolina State University, Raleigh, NC USA; 3 Comparative Medicine Institute, North Carolina State University, Raleigh, NC USA; 4 Collaborations Pharmaceuticals, Inc., 840 Main Campus Drive, Lab 3510, Raleigh, NC 27606 USA

**Keywords:** Computational biology and bioinformatics, Drug discovery

## Abstract

Macrolactones, macrocyclic lactones with at least twelve atoms within the core ring, include diverse natural products such as macrolides with potent bioactivities (e.g. antibiotics) and useful drug-like characteristics. We have developed MacrolactoneDB, which integrates nearly 14,000 existing macrolactones and their bioactivity information from different public databases, and new molecular descriptors to better characterize macrolide structures. The chemical distribution of MacrolactoneDB was analyzed in terms of important molecular properties and we have utilized three targets of interest (*Plasmodium falciparum*, Hepatitis C virus and T-cells) to demonstrate the value of compiling this data. Regression machine learning models were generated to predict biological endpoints using seven molecular descriptor sets and eight machine learning algorithms. Our results show that merging descriptors yields the best predictive power with Random Forest models, often boosted by consensus or hybrid modeling approaches. Our study provides cheminformatics insights into this privileged, underexplored structural class of compounds with high therapeutic potential.

## Introduction

**Figure 1:**
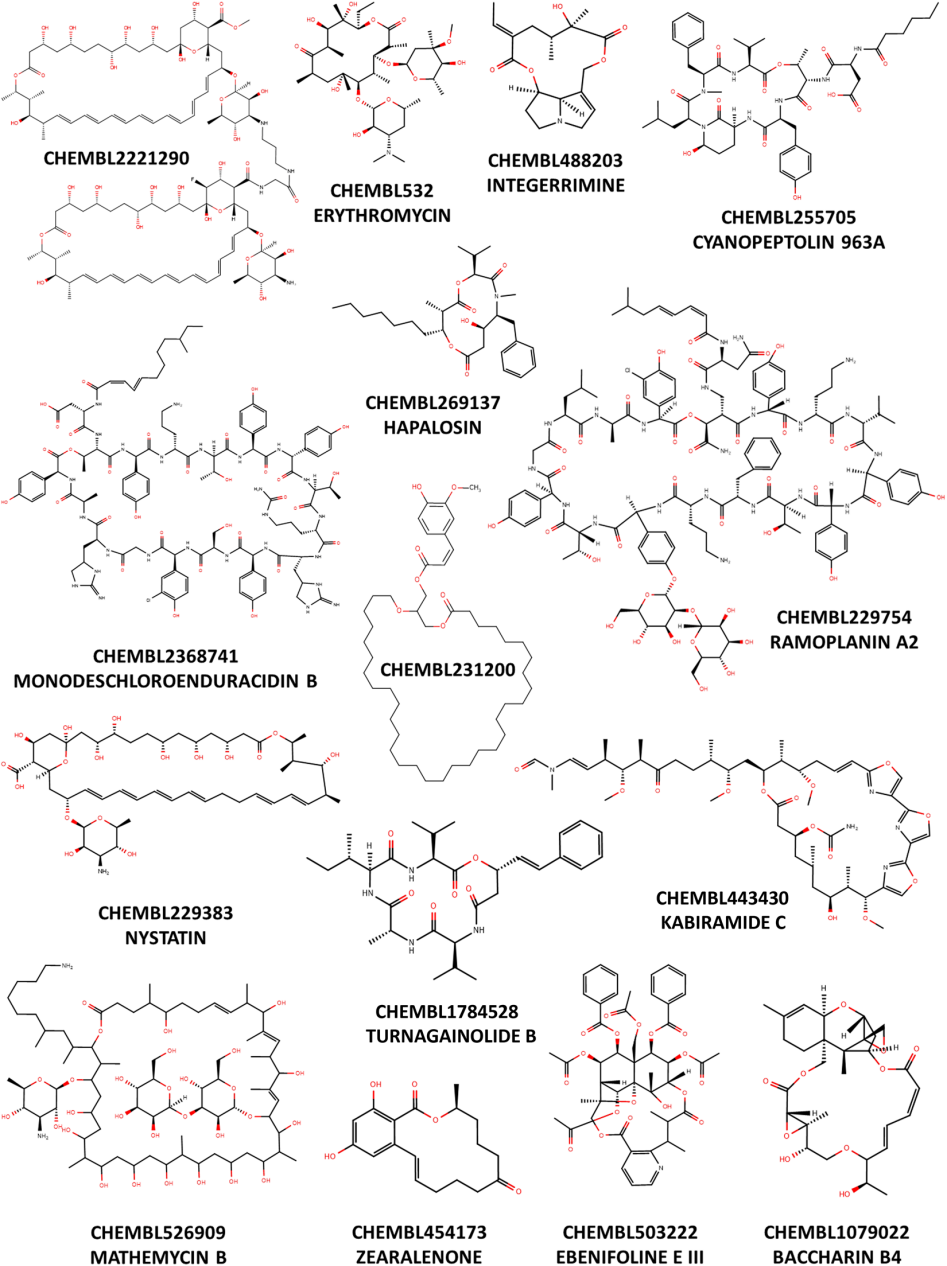
Example of macrolactones from MacrolactoneDB, along with associated names.

Macrocycles are at least 12-membered ring structures^1,2^. Of particular interest are macrolides and macrolactones, a privileged structural class commonly found in bioactive natural products^3,4^ and widely researched in pharmaceutical drug discovery^1–3,5–9^ (Fig. 1).

The design of macrocyclic drugs has been inspired by naturally occurring polyketides, secondary metabolites in certain living organisms^7^. These molecules clearly violate druglike rules such as the ‘Rule of 5’^10^ but they have been successfully employed as antiviral, antibiotic, antifungal, antiparasitic agents^11^ (e.g. erythromycin, clarithromycin, azithromycin). Macrocyclic structures are also interesting because of their ability to bind to difficult, undruggable protein targets, and display unusual physicochemical properties^5^. Hence, studying this structural class could yield important findings to help identify essential characteristics for novel macrolactone drug design. Distinguishing aspects of cyclic drugs are their rigidity which reduces undesirable side effects, the associated entropic costs to increase binding affinity, stability to proteolytic degradation, ability to bind to difficult targets with large binding pockets^5–7,12^ and ‘chameleonic’ ability to flip conformations. They also tend to have higher membrane permeability and metabolic stability^6,7^.

Despite this, macrolactones have been underexploited due to structural complications and difficult organic synthesis. Traditional organic synthetic approaches towards macrocyclic compounds have proven extremely challenging, usually involving numerous steps. Chemical databases such as ChEMBL^13^, PubChem^14^, ZINC15^15^ are indispensable to computer-aided drug discovery (CADD). They provide valuable biological/chemical information to build structure-activity relationship (SAR) models for screening, discovering and designing new drugs. However, there is no specialized large database for known, existing macrolactones in the public domain to our knowledge and this impedes the exploration and understanding of macrolactones. Specifically, a database of macrolactones has the potential to inspire the development of new drugs of this class.

Hence, one major goal of this study was to develop MacrolactoneDB. In this study, we mined macrolactones from public repositories; NANPDB^16^, StreptomeDB^17^, unpd^18^, NuBBe^19^, ZINC15^15^, TIPdb^20^, AfroDB^21^, BindingDB^22^, AfroMalariaDB^23^, BIOFACQUIM^24^, ChEMBL^13,25^ and PubChem^14^ along with available biological information extracted from ChEMBL^13^ (Table S1**, graphical abstract**) to create a database of macrolactones with bioactivities and a front-end web application.

Macrolactones are a broad, diverse structural class with various levels of complexity, and the database of macrolactones needs to be useful for different research projects. For example, biosynthetic chemists who work closely with “classic” macrolides such as erythromycin, pikromycin, etc. may be interested in a very specific set of twelve-to-sixteen-membered macrolides with sugars present. Additionally, the definition of “macrolides” has evolved in the past 50 years due to developments in medicinal research such as first, second and third generation of macrolides, ketolides, etc.^4,26^. Thus, to accommodate research groups focusing on different areas of macrolactones, we constructed a web application with multiple filters on chemical properties such as ring size, number of sugars, molecular weight, etc. to allow users to extract a highly specific subset of interest.

Additionally, we conducted a cheminformatics analysis of MacrolactoneDB to better understand the chemical diversity and scope of this structural class. We analyzed the chemical distribution in terms of several important molecular properties: molecular weight (MW), polar surface area (PSA), hydrophobicity (SlogP), hydrogen bond donors (HBD), hydrogen bond acceptors (HBA), rotatable bonds (NRB), and ring size (RS). To further demonstrate the chemical diversity and scope of macrolactones, we visualized the chemical network of MacrolactoneDB and incorporated biological activities in the form of pChEMBL into the network.

**Figure 2:**
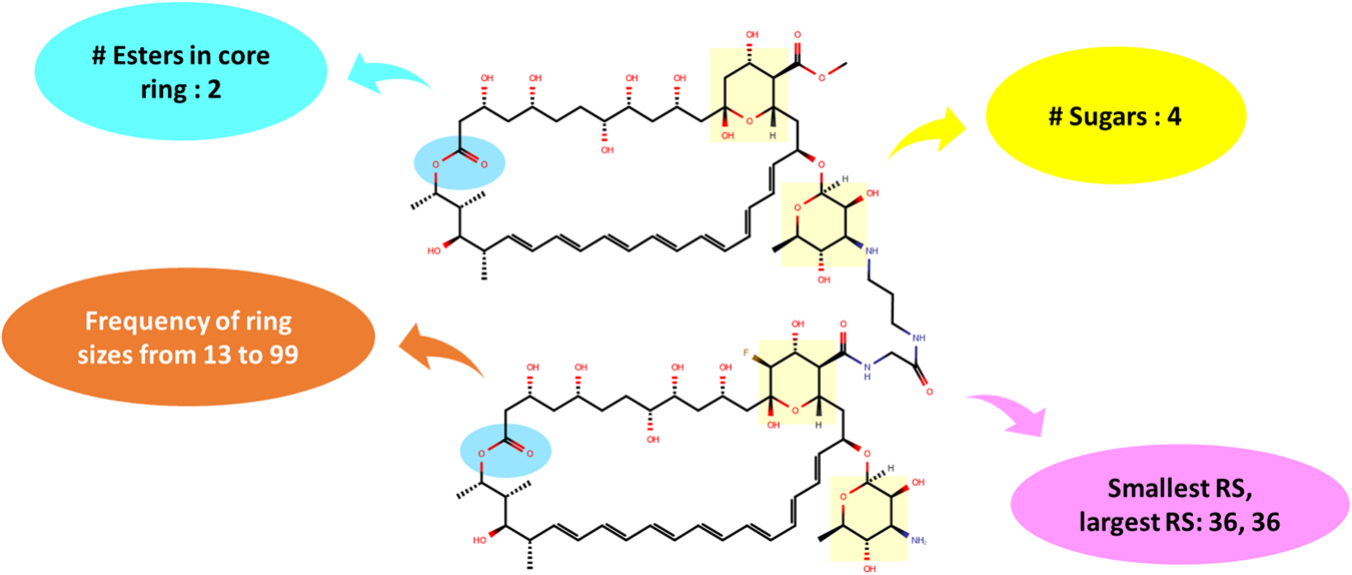
Illustration of mrc descriptors with CHEMBL2221290 as an example structure. Descriptors include frequency information on ring sizes of 13 to 99, largest and smallest ring sizes, number of sugars and number of esters in the core ring structures.

We also observed that contemporary chemical descriptors or fingerprints lack information to sufficiently account for large bioactive ring structures such as frequency of ring sizes larger than twelve, sugars etc. Thus, they may not fully characterize macrolactone molecules. Consequently, the lack of these important details can adversely affect Quantitative Structure-Activity Relationship (QSAR) and Mechanism of Action (MoA) studies. Thus, we developed 91 new descriptors (mrc) (Fig. 2), which account for frequency of ring sizes ranging from thirteen to ninety-nine, sugars, core esters, etc. to better characterize macrolactones and macrolides.

As the main case study, we extracted the three most common targets from MacrolactoneDB (*Plasmodium Falciparum* (malaria), Hepatitis C and T-cells), conducted cheminformatics analysis on the associated macrolactone ligands, and developed our machine learning (ML) workflow. QSAR modeling was conducted using a variety of state-of-the-art ML algorithms and molecular descriptor sets. We applied 10-fold cross validation (CV) on our three case studies and examined the relevance and usefulness of different cheminformatics methods and tools on these highly complex, large ring molecules. Our overarching goal for such modeling was to determine the optimal combination of ML algorithm and fingerprint set, and to provide chemical insights into macrolactones. Our workflow (Fig. S1) uses contemporary ML algorithms such as Random Forest (RF), Support Vector Regression (SVR), Naïve Bayes (NB), K nearest neighbor (KNN), Deep Neural Nets (DNN), Consensus (CSS, averaged endpoint among all the aforementioned ML algorithms), and Hybrid approaches from the two best algorithms (RF_KNN – average prediction from RF and KNN, and RF_ DNN – average prediction from RF and DNN). Our workflow utilizes explicit and implicit molecular descriptors which include mordred^27^, mrc (newly developed descriptors to address macrolide characteristics), mordred_mrc, MACCS, ECFP6, 2Drdkit, and “all” (a merger of unique, aforementioned descriptor sets).

## Results

### Analysis of macrolactoneDB

To assess the chemical diversity of molecular properties displayed by macrolactones, we analyzed MacrolactoneDB by studying the distribution of important molecular properties (MW, SlogP, TPSA, HBA, HBD, NRB, RS). These properties, except RS, are known to have a significant influence on bioavailability and membrane penetration^28^, and are commonly used to assess drug likeness, bioavailability, and oral absorption according to Lipinski’s ‘Rule of 5’ and Veber’s rules^28,29^. The descriptive statistics regarding these molecular properties are shown in Table S2.

**Figure 3:**
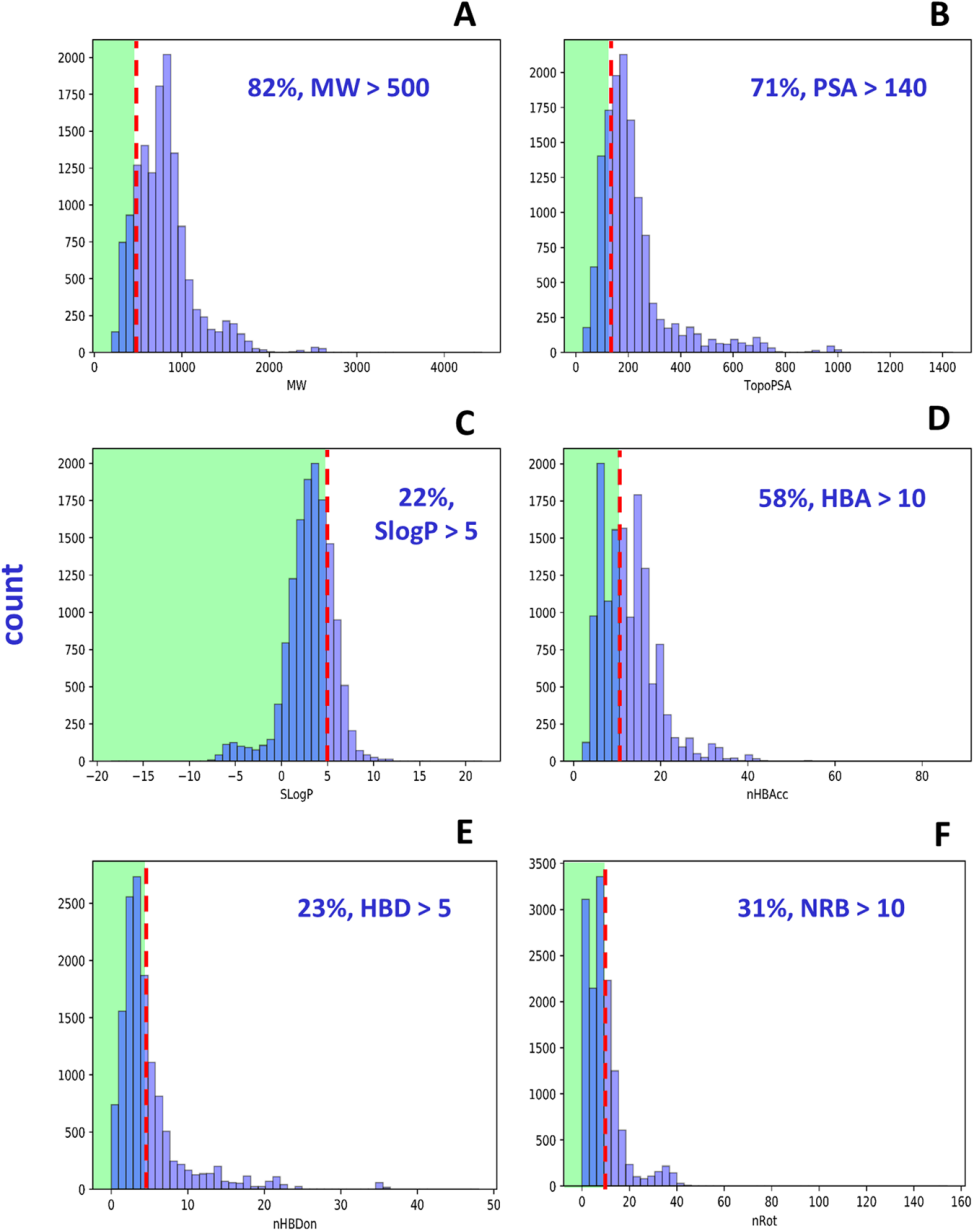
Distribution of molecular properties: (**A**) Molecular Weight - MW, (**B**) Polar Surface Area - PSA, (**C**) Hydrophobicity - SlogP, (**D**) Hydrogen Bond Acceptors - HBA, (**E**) Hydrogen Bond Donors - HBD, (**F**) Number of Rotatable bonds - NRB of MacrolactoneDB. Green rectangular areas show druglike and bioavailable regions according to Lipinski’s Rule of 5 and Veber’s rules.

MW of macrolactones followed a right-skewed distribution and ranged from 194 to 4429 g mol^−1^ with a mean of 787 ± 339 g mol^−1^ (Fig. 3A, Table S2). An overwhelming 82% exceeded MW of 500. PSA followed a right-skewed distribution ranging from 26.3 to 1439 °A^2^ with a mean of 213 ± 139 °A^2^ (Fig. 3B, Table S2). Almost 71% exceeded PSA of 140. SlogP followed a bell-shaped distribution with a range from −18.5 to 21.7, and a mean of 3.10 ± 2.65 (Fig. 3C, Table S2). Interestingly, less than 22% exceeded SlogP of 5.

HBA followed a right-skewed distribution and ranged from 2 to 87 with a mean of 12.7 ± 6.36 (Fig. 3D, Table S2). More than half (58%) violated HBA of at most 10. HBD followed a right-skewed distribution with a range from 0 to 48 and a mean of 4.63 ± 4.88 (Fig. 3E, Table S2). 23% disobeyed HBD of at least 5. NRB followed a right-skewed distribution and ranged from 0 to 154 with a NRB_mean_ of 9.21 ± 7.98 (Fig. 3F, Table S2). 31% surpassed NRB of 10.

RS ranged widely from 12 to 55 with a mean of 17.4 ± 5.99 (Fig. S2, Table S2). 14-membered macrolactones are the most common in MacrolactoneDB, representing 22.3% (3056 compounds) of the database. 12 to 16-membered macrolactones, the common RS range for classic macrolides represent ~65% (8914 compounds) of the entire database. 11,487 compounds (84%) of macrolactones were found to violate Lipinski’s Ro5^29^.

**Figure 4:**
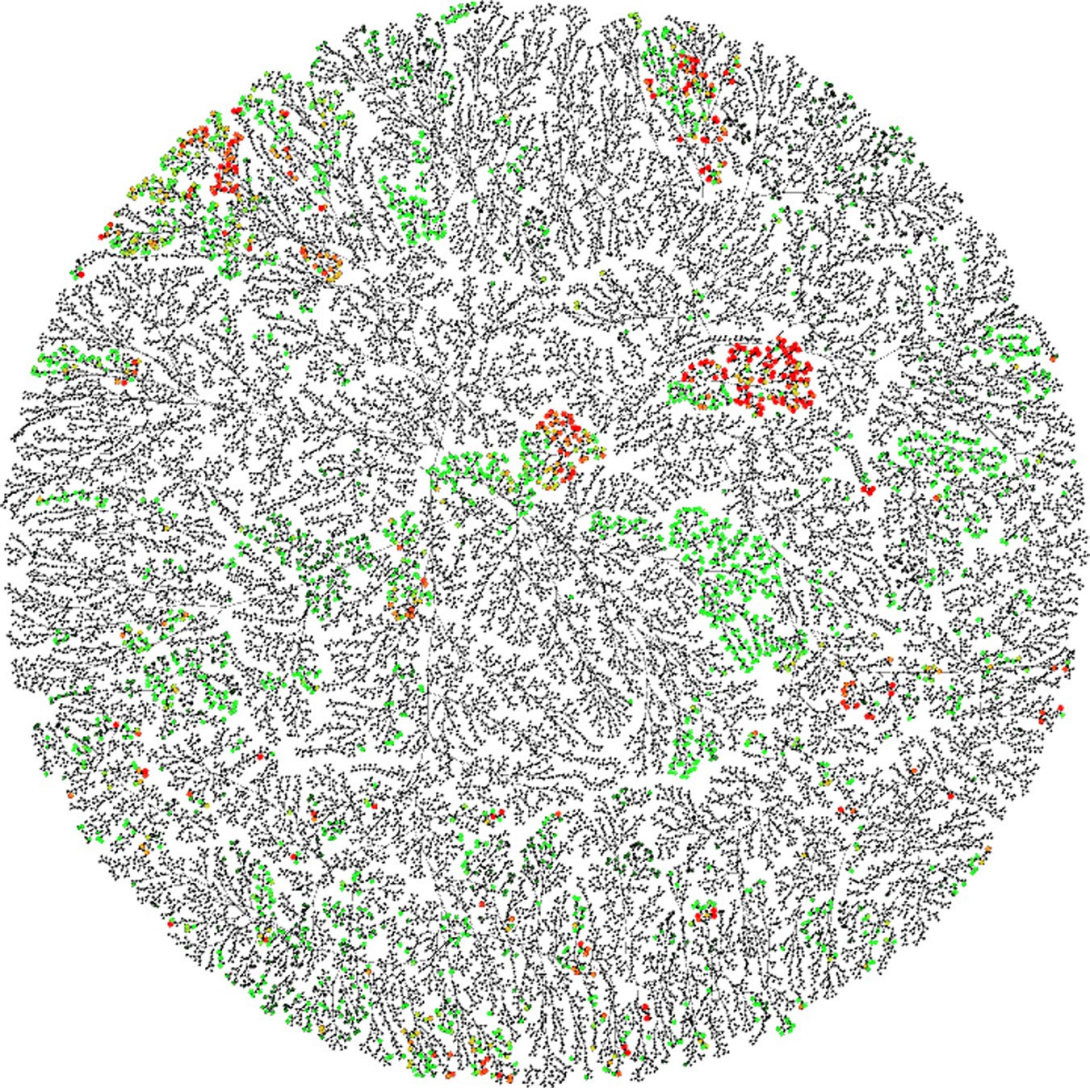
Chemical Space Visualization of MacrolactoneDB; color and size represent compounds with pChEMBL values where red indicates low pChEMBL values and green high pChEMBL values. Compounds with unreported pChEMBL values are colored black.

We visualized the chemical network of MacrolactoneDB (Fig. 4**)** and incorporated their bioactivities. Overall, MacrolactoneDB had a comprehensive collection of large ring structures with different degrees of complexity and covered a large scope of chemical properties with widely varying ranges.

### Relevance of rule of 5 to bioactivites of macrolactones

We assessed the relevance of Lipinski’s Rule of 5 to the reported bioactivities of macrolactones in the general activity spectrum. We assessed the maximally reported pChEMBL values of macrolactones and observed whether they obeyed or violated the Rule of 5. Compounds with the maximal pChEMBL value of at least 7 were considered active and those below 7 inactive. Fig. S3 shows the summary statistics on the relevance to the Rule of 5 with respect to the activity of macrolactones, along with scatter plots of MW and SlogP, MW and TopoPSA. Among all activities reported for macrolactones, 455 (17%) abided by these rules among which only a very small population of 91 (3%) which were active. Many pChEMBL reported macrolactones, approximately 2300 (83%), violated the Rule of 5 yet a comparatively large population of ~1100 (41%) were found active.

### Distribution analysis of biological endpoints of macrolactone ligands

In QSAR modeling, pIC50s (negative log of IC_50_; concentration of inhibitor at 50% of the total inhibition^30^) were used as prediction endpoints for *Plasmodium falciparum* and T-cells targets. However, pIC50s for the Hepatitis C target had very limited variation and highly skewed distribution (not reported); thus, a mix of pIC50 and pEC50 (negative log of EC_50_; the effective concentration at 50% of the total effect^30^) were used as biological endpoints in building our QSAR regressors. For compounds with both pIC50 and pEC50 reported, we only used pIC50 since the dataset predominantly had IC_50_’s reported.

The distribution of pIC50 for *Plasmodium falciparum* - Malaria (CHEMBL364), pIC50/pEC50 for Hepatitis C targets (CHEMBL379) and pIC50 for T-cells (CHEMBL614309), were shown in Fig. S4A–C respectively. Malaria had 223 macrolactone ligands with an almost normal distribution of pIC50s ranging from 4.67 to 8.61 and a mean of 6.59 ± 0.65. Hepatitis C had 129 macrolactone ligands with a slightly left-skewed distribution of pIC50/pEC50s ranging from 4.48 to 9.59 and a mean of 7.29 ± 1.04. T-cells had 103 macrolactone ligands with an abnormal distribution of pIC50s ranging from 4.93 to 9.74 and a mean of 8.24 ± 1.07. The box plot distribution showed the Hepatitis C dataset covered the widest, most well-distributed range of pIC50/pEC50 values whereas the other two had relatively narrower pIC50 distributions (Fig. S4D). Overall, all the datasets had a good range of activities suitable for QSAR regressors.

### Cluster analysis of macrolactone ligands

We conducted an unsupervised hierarchical clustering using Euclidean distance, Ward Linkage with (A) mordred_mrc, and (B) ECFP6 (2048 bits) using ggtree package^31^ (v1.10.5) in R (v3.4.4). The resulting circular dendrograms for *Plasmodium falciparum*, Hepatitis C and T-cell datasets were provided in Figs. S5–S7 respectively. Each dendrogram node was colored according to the experimental pIC50 or pIC50/pEC50 values afforded by the corresponding chemical structure. This procedure allowed us to identify analogues having similar structures or chemical properties. Additionally, it could point to the importance or relevance of descriptors based on whether they can cluster compounds with similar activities.

In the *Plasmodium falciparum* dataset, we identified two interesting clusters with mordred_mrc (Fig. S5A) whose members were clustered apart based on ECFP6 (Fig. S5B). The first cluster in mordred_mrc contained two macrolactones with varying pIC50 values (red arrows in Fig. S5A). Their structures were shown in Fig. S5C and different structural components were highlighted yellow. This suggests these two compounds share similar chemical properties, but different structural fragments which contributed to varying IC_50_’s. In fact, they had a Tanimoto coefficient (similarity measurement between two chemicals^32^) of 0.87 (MACCS-166 bits) and a difference of 11,900 nM in their IC_50_’s. This is an example of activity cliff, where “large” differences in potency were observed despite two structures having “similar” structures^33^, identified by mordred_mrc. These analogs are interesting because it sheds light on chemical components of interest in macrolactone structures that play an important role in bioactivities and thus are worth exploring and manipulating for future SAR studies and for improving potencies.

Similarly, in another cluster, three compounds with similar pIC50s, clustered together with mordred_mrc (blue arrows in Fig. S5A) were clustered apart with ECFP6 (blue arrows in Fig. S5B). Their structures were shown in Fig. S5D, differing structural components highlighted, and shared structural fragments indicated with color-coded star symbols. They had the same core ring structure, but side chains were modified at N10 or in the sugar component at C5 (Fig. S5D). Regardless of these structural differences, they were similar in terms of chemical properties and in pIC50s. These analogs are also interesting since they showed the impact of structural modification on biological endpoints towards *Plasmodium falciparum*. In this cluster, modifying the sugar component (the side chain in tertiary amino of CHEMBL1946558) yielded higher bioactivity among all analogues. Merging the structures of CHEMBL1946558 and CHEMBL2029590 could be of interest but also could impose a synthetic challenge. Structural modifications at these different positions may lead to more interactions with the target and could perhaps yield higher bioactivity unless the sidechains occupy identical binding sites. In both clusters based on mordred_mrc, we identified highly similar structural analogues with a few modifications in the side chains affording different pIC50s. This cluster analysis demonstrates that we can extract useful structure-activity relationships and study the influence of minor structural changes in macrolactones on their activities which can inturn lead to the design and investigation on new compounds of interest. Additional cluster analysis regarding Hepatitis C and T-cells case studies is provided (**Results S1)**. Further, the distribution analysis of mrc properties for macrolactone ligands of these three case studies is provided (**Results S2)**.

### Assessment and comparison of descriptors and machine learning algorithms

**Figure 5:**
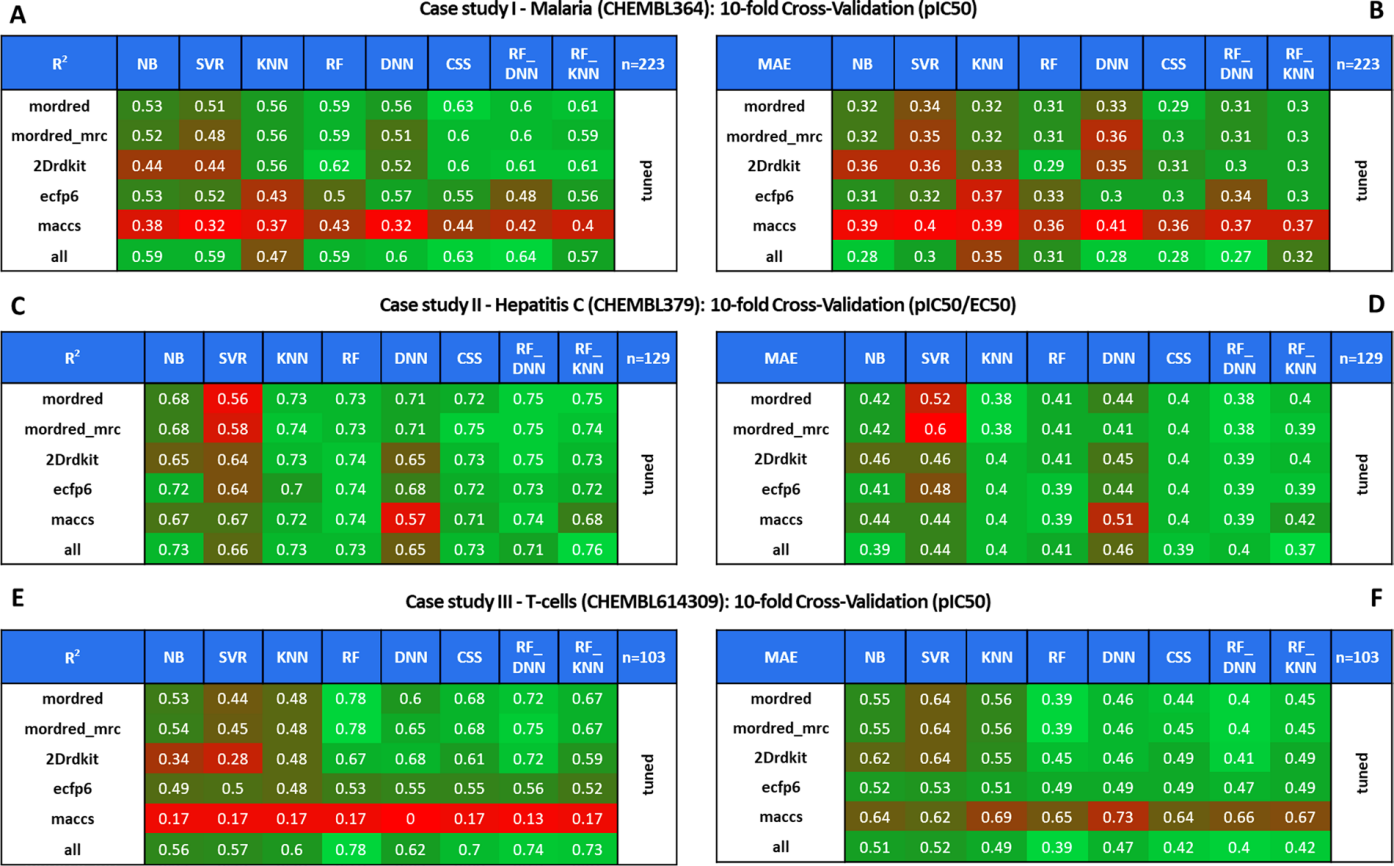
10-fold Cross-Validation across eight machine learning algorithms and six fingerprint/descriptor sets with tuned method for (**A**) R^2^ (coefficient of determination) for *Plasmodium falciparum*, (**B**) MAE (mean absolute error) for *Plasmodium falciparum*, (**C**) R^2^ for Hepatitis C, (**D**) MAE for Hepatitis C, (**E**) R^2^ for T-cells, and (**F**) MAE for T-cells.

We compared the performance of molecular descriptors and ML models from tuned methods for all our cases studies. Heatmaps with statistics on R^2^ and MAE results for *Plasmodium falciparum*, Hepatitis C and T-cell case studies with tuned methods can be found in Fig. 5. More details on R^2^ and MAE for the entire QSAR modeling workflow including base and pca_tuned methods are provided in Figs. S10–S12 and the impacts of tuning parameters and feature extraction techniques on QSAR modeling is described in Results S3. To determine the optimal set of descriptors, we generated clustered columns across 8 MLs for each descriptor set in R^2^ (Fig. S16) and MAE (Fig. S17).

In the *Plasmodium falciparum* dataset, “all” descriptors provided the best prediction results consistently across 8 MLs based on R^2^ (highest, Fig. S16A), and MAE (lowest, Fig. S17A). On the other hand, MACCS, a structural fragment-based fingerprint approach, had the lowest performance by comparison with R^2^_max_ = 0.44 (Fig. S16A) and MAE_min_ = 0.36 (Fig. S17A). In general, implicit fingerprints; ECFP6 (R^2^_max_ = 0.57, MAE_min_ = 0.30) and MACCS, did not perform as well as explicit descriptors or the merger “all”.

In the Hepatitis C dataset, the descriptor sets had very similar R^2^ and MAE across 8 MLs; with “all” descriptors slightly better with R^2^_max_ 0.76 (Fig. S16B) and MAE_min_ 0.37 (Fig. S17B). The T-cells dataset results showed a significant difference in the performance between explicit and implicit descriptors. Explicit descriptors (mordred, mordred_mrc and merger “all”) had R^2^_max_ 0.78 (Fig. S16C) and MAE_min_ 0.39 (Fig. S17C) across MLs. On the other hand, implicit fingerprints; ECFP6 (R^2^_max_ = 0.56, MAE_min_ = 0.47) and MACCS (R^2^_max_ = 0.17, MAE_min_ = 0.62) yielded very poor performances. Our QSAR models with MACCS did not find any meaningful correlation for macrolactone ligands of the T-cells target with any ML algorithms.

Overall, the prediction results from the case studies agreed with our hypothesis that explicit descriptors (mordred, mordred_mrc) would perform better than implicit fingerprints (MACCS or ECFP6); especially in Malaria and T-cells case studies. Of note, “all” descriptors performed best among others, closely followed by either mordred or mordred_mrc in all cases. In fact, the difference of highest R^2^ and lowest MAE across MLs between “all” and mordred/mordred_mrc were 0.01 and 0.02 at most respectively. Thus, adding MACCS or ECFP6 to mordred_mrc only slightly affected the predictive power of QSAR models. Perhaps, it is sufficient to use mordred or mordred_mrc alone to build regression models; however, there is no harm in using “all” descriptors to account for property-related and structural variations captured by both explicit and implicit descriptors. Afterall, across our case studies, “all” descriptors provided slightly superior performance; if not equal to mordred or mordred_mrc descriptors.

**Figure 6:**
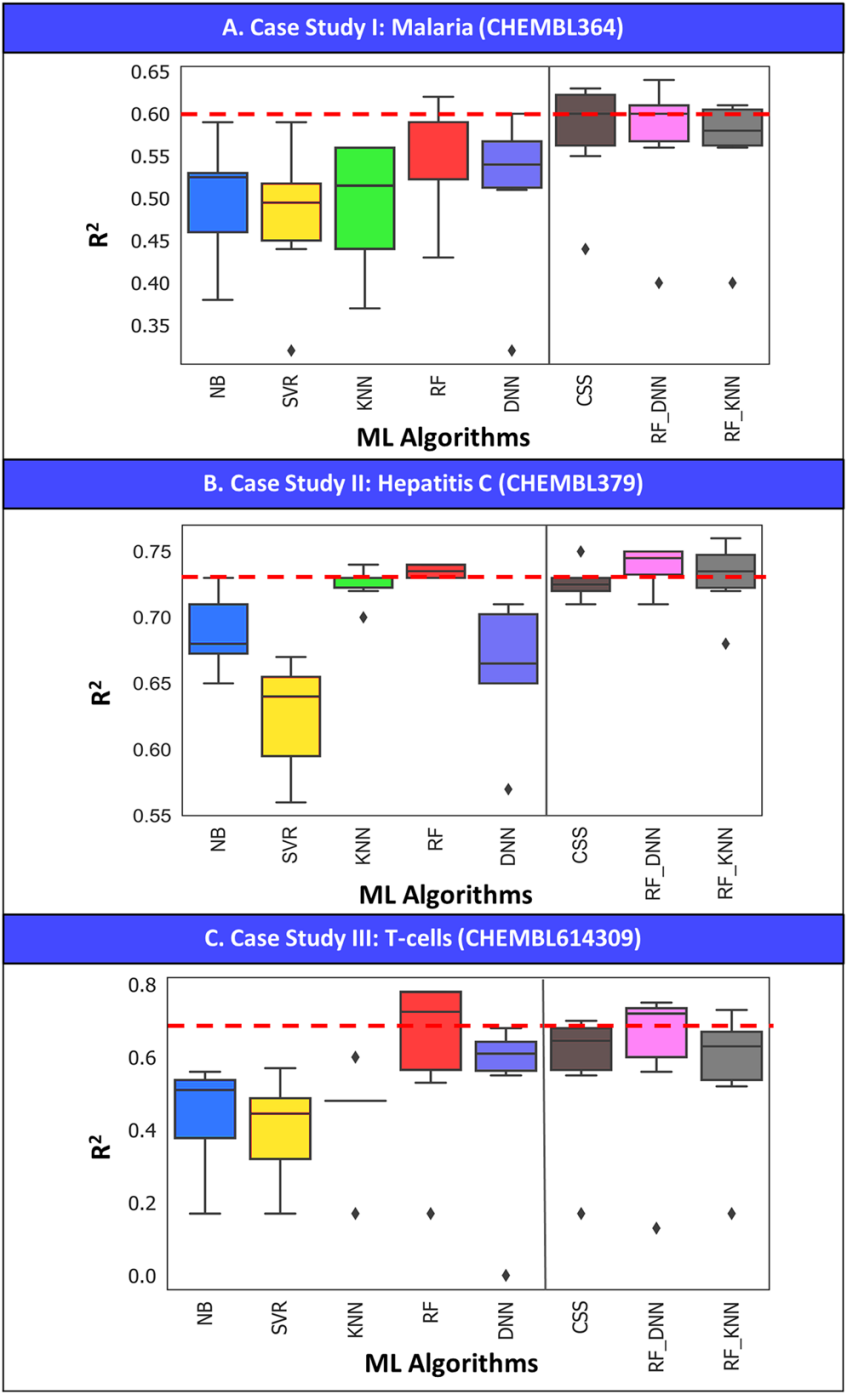
Boxplot analysis of ML algorithms with associated R^2^ distribution across six descriptor sets with tuned method for (**A**) Plasmodium falciparum, (**B**) Hepatitis C Virus, and (**C**) T-cells.

When consensus and hybrid modeling approaches were applied, we noticed an increase in R^2^ across our case studies, especially for *Plasmodium falciparum* (Fig. 6A) and Hepatitis C (Fig. 6B). In the *Plasmodium falciparum* case study (Fig. 5A for R^2^ and Fig. 5B for MAE), RF_DNN afforded R^2^_max_ 0.64 and MAE_min_ 0.27 with “all” descriptors whereas RF alone afforded the R^2^_max_ 0.62 and MAE_min_ 0.29 with 2DRDKit descriptors. Moreover, CSS achieved consistently high R^2^ ~ 0.63 across descriptors except for ECFP6 and MACCS.

In Hepatitis C, RF_DNN steadily afforded high R^2^ 0.75 (Fig. 5A) across descriptors and low MAE (Fig. 5B**)** 0.38, closely matched by RF_KNN and CSS. In T-cells, RF individually was still the best among 8 MLs including CSS, RF_DNN and RF_KNN (Fig. 6C). However, CSS, RF_DNN and RF_KNN closely matched the performance of RFs and were superior to other individual ML algorithms including DNN.

Lastly, we sampled a few unique, outlier macrolactones from each case study either based on ring size, structural complexity, and assessed the performance of ML 10-fold CV on them. **Results S4** showed that we still afforded excellent predictions on such complex, uniquely ring-sized molecules even though the training set barely covered the chemical scope of these structures.

### Y-randomization

We further validated and eliminated the possibility of chance correlation in our QSAR models by conducting y-randomization with 10-fold CV for the three case studies. In this process, we scrambled target responses in the training folds in 10-fold cross-validations with six descriptors (2Drdkit, “all”, ECFP6, MACCS, mordred, mordred_mrc) and 5 MLs (NB, SVR, KNN, RF, RF_KNN). The entire process of y-randomization with 10-fold cross-validation was repeated 10 times. This workflow should suffice in establishing whether there is a presence of chance correlation in our QSAR modeling, and we did not perform DNN since it would require extensive computational resources. We evaluated our y-randomized models using the coefficient of determination (R^2^). The maximum R^2^ from 10 iterations of y-randomization study were reported in Tables S6–S8 for Malaria, Hepatitis C virus, and T-cells datasets respectively. The highest y-randomized R^2^ for the combination of ML and descriptor set afforded were 0.11 (≪0.64 from actual QSAR 10-fold CV R^2^) for Malaria target, 0.14 (≪0.76 from actual QSAR 10-fold CV R^2^) for the Hepatitis C virus target, 0.18 (≪0.78 from actual QSAR 10-fold CV R^2^) for the T-cells target. All these maximally achieved R^2^ from the best optimal combination are obviously well below the R^2^ afforded by our actual QSAR models, thus it rules out the possibility of chance correlation for our QSAR models.

## Discussion

MacrolactoneDB provides a comprehensive database of curated macrolactones with bioactivities across many targets. Such a database is likely of interest to researchers and presents opportunities for repurposing these compounds for new uses. Compounds from MacrolactoneDB can be used as scaffolds that can be manipulated and modified to design novel macrolides using biosynthetic engineering methods, semi-synthesis or traditional organic chemistry. Ergo, it can motivate the development of novel polyketide antibiotics, and encourage further exploration of challenging, underexplored macrolactones. We believe MacrolactoneDB will launch a plethora of cheminformatics studies including QSAR. For example, chemical insights from conducting frequency analysis of structural fragments, i.e. building blocks, in MacrolactoneDB can be used in conjunction with other software such as PKS Enumerator^34^ or SIME  to effectively design large in-silico libraries of macrolides with improved drug-likeness and synthetic feasibility.

We conducted a cheminformatics analysis of MacrolactoneDB to understand the chemical scope and coverage of these large ring molecules. An overwhelming majority of them evidently violated Lipinski’s ‘Rule of 5’ and Veber’s rules of drug likeness and oral bioavailability^10,35^. However, the assessment of their bioactivity showed that they still afforded interesting bioactivities despite their violations. In fact, a much larger number of macrolactones beyond the Rule of 5 were found to be biologically active than those within. That was perhaps due to the “chameleon-like” behavior of macrocyclic structures; the ability to change shape, polarity, or conformation driven by intra-molecular hydrogen bonding based on its surrounding aqueous or non-polar environment^10,36^. The analysis of MacrolactoneDB signifies an urgent need to develop new drug likeness and bioavailability rules specific to medium to large sized molecules such as these macrolactones.

Additionally, we developed mrc descriptors to better characterize macrolactones, provide insight into their SAR and improve predictive modeling with integration to other descriptors such as mordred. The usefulness of mrc descriptors will be proven in chemical datasets with large variance, well-distributed ring size and macrolide related characteristics. One feature in mrc was the frequency of sugars, a crucial component influencing the bioactivities of macrolides and contributing one-half to two-thirds of the binding energy^37^. Classic macrolide structures, usually twelve to sixteen-membered rings^4,38^, have one or more sugars attached to the core rings via glycosidic bonds. We hypothesize the presence/frequency of sugars could thus be important in characterizing macrolactones/macrolides. However, specific sugar types such as cladinose, desosamine, etc. are not yet recognized by mrc; only the total frequency of sugars. Examining chemical databases, mining sugar components, identifying and incorporating various types of sugars to mrc will certainly be worth exploring. Information on the occurrence of specific sugar types and atomic positions for glycosidic bonds will also be helpful in extracting SAR relationships and building predictive models for macrolides.

We demonstrated in our proof-of-concept study with three common disease targets that machine learning models for macrolactones/macrolides can be trained and validated using various algorithms and descriptors. We conducted QSAR modeling with macrolactones on *Plasmodium falciparum*, Hepatitis C virus and T-cells from MacrolactoneDB with eight MLs and seven descriptor sets. Since large structures such as macrolactones are underexplored in QSAR modeling, the insights from this study could be valuable to the cheminformatics community. It is, in fact, one of the first such studies exploring and tackling this structural class of macrolactones by applying multiple cheminformatics techniques such as several combinations of ML algorithms and chemical descriptors.

Our three case studies showed that RF was the best predictor among individual ML algorithms across six descriptor sets. We also demonstrated that consensus modeling from five ML algorithms or hybrid approach which averages the prediction results from two ML algorithms (in this case study, RF_DNN and RF_KNN) slightly increased the predictive power of QSAR models built with individual ML algorithms.

Regarding the descriptors, we expected mordred, mordred_mrc or “all” would be most useful and relevant in predicting the bioactivities since they would convey a more comprehensive representation of macrolactone structures. We also applied implicit chemical fingerprints (ECFP6, MACCS) to assess and compare the performance of structural fragment-based approach in predicting the macrolactone bioactivities. Our results from all three case studies showed that the merger “all” was the best feature types for macrolactones, followed closely by mordred and mordred_mrc descriptors across MLs, which agreed well with our initial expectation. An interesting observation was that explicit descriptors outperform implicit descriptors across MLs and case studies, except in the case of Hepatitis C wherein implicit and explicit descriptors performed equivalently. Mordred_mrc was the most useful set of descriptors; even merging other descriptor sets such as ECFP6 and MACCS to mordred_mrc (“all”) did not make a significant improvement in comparison to mordred_mrc alone.

The analysis of macrolactone ligand datasets in the three case studies showed narrowly distributed and limited variation of macrocycle related properties addressed by mrc descriptors (**Results S2**), thus they are likely not ideal datasets to assess the true performance of mordred_mrc descriptors. Understandably, we did not see any noticeable difference in the performance of mordred and mordred_mrc across MLs.

One important finding was our QSAR models’ ability to predict highly accurate biological endpoints on highly complex, uniquely ring-sized (‘misfit’) macrolactones relative to the rest of the datasets in 10-fold CV (**Results S4**). We demonstrated the high predictive performance of QSAR models affording “top” predictions on larger, more complex, unique macrolactones even when they were trained with dominantly different-sized ring structures. This highlighted the usefulness of contemporary chemical descriptors and ML algorithms.

Of note, this workflow was built with only 2D fingerprints/descriptors, thus the information characterizing these macrolactones may not capture the conformational information such as intra-hydrogen bonding properties. Yet, the predictive power of these models built with 2D descriptors alone was rather impressive (R^2^ = 0.64 for Plasmodium falciparum, R^2^ = 0.76 for Hepatitis C Virus, R^2^ = 0.78 for T-cells). Conformational analysis of macrocycles still remains a complex, challenging problem wherein a small structural modification can result in conformational reorganization of remote regions of a macrocyclic backbone^36^. It is hoped that when such information is included the models will improve further. However, our study confirmed 2D-descriptor-based QSAR models developed with compounds from MacrolactoneDB can be used to predict biological activities of new macrolides and to prioritize potential biosynthesis. This will have value in the search for novel macrolactone/macrolide therapeutics.

## Methods

### MacrolactoneDB

MacrolactoneDB is a web application hosting ~13,700 macrolactones, including macrolides mined from public repositories such as NANPDB^16^, StreptomeDB^17^, unpd^18^, NuBBe^19^, ZINC15^15^, TIPdb^20^, AfroDB^21^, BindingDB^22^, AfroMalariaDB^23^, BIOFACQUIM^24^, ChEMBL^13,25^ and PubChem^14^. Ring structures with at least twelve members and ester functional group(s) within the core rings were filtered using *RDKit Molecule Substructure Filter* node and SMART patterns in Knime^39^. The detailed information regarding the number of compounds for each database is available (Table S1). All filtered structures were curated using a similar protocol proposed by Fourches *et al*.^40^; 1) removal of mixtures, inorganics, 2) structural conversion, cleaning/removal of salts, 3) structural normalization, and 4) removal of structural duplicates. This resulted in currently 13,721 diverse macrolactones in MacrolactoneDB at http://macrolact.collabchem.com/. Example chemical structures were randomly picked from MacrolactoneDB (Fig. 1). All the available biological activity information was retrieved from ChEMBL database using ChEMBL web services^41^ python 3.6. Due to the large structural diversity of macrolactones, we developed a user-friendly web interface with filters that allow users to restrict and subset the chemical space of interest (**Methods S1**).

### Macrolide related (mrc) descriptors

We developed 91 macrolide-related descriptors to better characterize macrolactones and to complement mordred descriptors^27^. mrc descriptors include information on frequency of ring sizes ranging from 13 to 99, smallest and largest ring sizes (≥12-membered), frequency of sugars and occurrence of esters within the core rings. An illustrative diagram of mrc descriptor along with an example structure of CHEMBL2221290 is shown in Fig. 2.

Mordred is, so far, the most comprehensive 2D descriptor set (approx. 1,600 features) which includes not only the complete set of RDKit descriptors^42^, but also accounts for ring structures with ring sizes up to 12. Hence, we developed mrc (macrolactones related) descriptors to complement mordred, and mrc accounts for the presence and frequency of ring sizes ranging from 13 to 99. The specified ring size in mrc should sufficiently cover the macrolactone space because the largest macrolide ever reported in the literature was Zooxanthellamide Cs with 63 to 66 atoms in the core ring structures^43^. The ring size-based features in mrc were built on top of Mordred descriptors.

One feature in mrc was the frequency of sugars based on six sugar SMART patterns as identified by cdk^44^ (**Table S3**). It was, however, unable to recognize specific sugar types as cladinose, desosamine, etc. thus it only accounted for the occurrence of sugars instead of individual sugar types. Another mrc feature is the count of esters in the core ring structures. Classic macrolides such as erythromycin, azithromycin have one ester within the core rings. The terminating thioesterase (TE) module usually found in the last domain in the type 1 NRPS multienzymes cleaves off the fully assembled peptide^45^, closes the ring structures, resulting an ester within the core ring. Thus, the count of core esters could be an important trait in determining the extent of bio-synthesizability for macrolides or a characteristic of natural products, and thus included as a feature in mrc descriptors.

The code to compute mordred_mrc descriptors has been provided in the github repository https://github.com/zinph/mordred_mrc. To assess the efficiency of mrc descriptors, we computed them for the entire MacrolactoneDB containing ~13,700 large, highly complex ring molecules with MW up to 4429.7 g.mol^−1^. This is perhaps the most challenging chemical dataset for computing complicated descriptors. Some calculators (e.g. PaDEL) result in missing values from time out^27^. mrc descriptor calculation for the entire MacrolactoneDB was completed in an acceptable time of 43 mins 38 s on an Intel Xeon W-2104 CPU, 32 GB memory machine whereas mordred descriptors took 1 hr 44 min 37 s. Further updates on algorithm optimization of mrc descriptors can be referenced in github repository.

Of note, mrc descriptors alone do not sufficiently characterize macrolactones since it only includes information on ring sizes and macrolide related characteristics. They were developed to complement mordred descriptors. For QSAR modeling, the importance and relevance of mordred_mrc descriptors will come into play for training datasets of macrolides with well-distributed properties addressed by mrc.

### Chemical network visualization of macrolactoneDB

Unsupervised hierarchical clustering was conducted with ECFP6 fingerprints calculated for the curated set of ~13,700 compounds in MacrolactoneDB using ggtree package^31^ (v1.10.5) in R (v3.4.4). The edges and nodes were generated based on Euclidean distance^46^ between the ECFP6 of the molecules and Ward’s minimum variance method linkage^47^ between clusters. We then generated a chemical network visualization of these compounds using Gephi 0.9.2^48^ and a combination of multiple layout algorithms such as MultiGravity ForceAtlas2, ForceAtlas2^49^, Contraction, Yifan Hu^50^, and Yifan Hu Proportional provided by the Gephi tool^48,51^. Each node in the chemical network was a macrolactone ligand (Fig. 4). The nodes in the chemical network were then colored according to the maximally reported pChEMBL value of the corresponding compound against any known target. For 1233 macrolactones, there were more than one pChEMBL value for the same ligand-target pairs, and there were multiple targets associated with 1479 macrolactone ligands. In this approach, we focused on the macrolactone ligands and their level in the general activity spectrum; thus, we chose only one target with any maximally reported biological endpoint. The color and size of the nodes represent pChEMBL values where green is associated with high and red is associated with low pChEMBL values (Fig. 4). The nodes for compounds with unreported pChEMBL values are colored black in the visualization network. Overall, only ~2,800 (20%) macrolactones had reported pChEMBL values (Fig. 4).

### Quantitative structure-activity relationship (QSAR) modeling

All the computing details can be found in **Methods S2**.

### Data curation

All the macrolactone ligands associated with three targets (ChEMBL364 – Plasmodium falciparum, ChEMBL379 – Hepatitis C virus and CHEMBL614309 – T-cells) were extracted and curated using the protocol proposed by Fourches *et al*.^40^. Only those with known pIC50 for Plasmodium falciparum and T-cells targets, and pIC50/EC50s for the Hepatitis C target were kept. For the same target-ligand pair, (1) only exact activity values were considered, (2) if there were several values of the same affinity type available, the average of all reported values within z-score of 2 were kept, and the rest treated as outliers and discarded. The full data sets are available in Supporting Information (Data S1).

### Descriptors and machine learning (ML) algorithms

In this study, we used the following fingerprint/descriptor sets: MACCS^52^ (166 bits), ECFP6^53^ (2048 bits), 2D RDKit^42^ (115 features), mordred^27^ (1613 features), mrc (91 features), mordred_mrc – a combination of mordred and mrc (1704 features), “all” – a combination of mordred_mrc, ECFP6 and maccs (3919 features).

The following ML methods were used in this study: RF, NB, SVR, KNN, DNN, CSS, RF_KNN and RF_DNN. These ML methods have been commonly and successfully used in cheminformatics studies, and summarized descriptions were provided in **Methods S3**.

### Overview and QSAR workflow development

We developed QSAR regression models by selecting three targets (Plasmodium falciparum, Hepatitis C and T-cells) with the most known macrolactone ligands with bioactivities reported. Our main objective in this QSAR modeling study is to assess the performance of contemporary 2D fingerprint/descriptor sets and ML algorithms on these underexplored large bioactive molecules.

We hypothesize explicit descriptors such as mordred or mordred_mrc would outperform implicit fingerprints such as MACCS, ECFP6 since structural fragment-based approach may not fully capture ring information as well as explicit descriptors. We also posit the frequency of RS and sugars would influence binding affinities; thus, adding mrc to mordred descriptors could boost the performance of QSAR models; especially if the training dataset has well-distributed properties addressed by mrc. However, different structural fragments undeniably play an important role in the chemical properties and interactions with the target proteins, thus we expect to see a certain level of importance and relevance in predicting their biological endpoints towards associated targets.

Overall, we explored the following cheminformatics-related questions. We assessed whether applying PCA or parameter tuning improved the predictive power of QSAR models. We explored whether explicit descriptors outperform implicit fingerprints, determined an optimal combination of ML and descriptor/fingerprint set that performs well on macrolactones, and assessed if mrc descriptors complement mordred descriptors and help boost the predictive power of QSAR models. Hence, we implemented the workflow which uses eight ML algorithms and seven descriptor sets (Fig. S1).

### QSAR model performance evaluation

To evaluate the performance of QSAR regression models, we used the following criteria: the mean absolute error (MAE), and the coefficient of determination (${R}^{2}$).

$$\begin{array}{c}MAE=\,\frac{{\sum }^{}|y-{y}_{pred}|}{n},\\ {R}^{2}=1-\frac{{\sum }^{}{(y-{y}_{pred})}^{2}}{{\sum }^{}(y-\bar{y})}\end{array}$$

where y = experimental pIC50, $\bar{y}$ = mean of experimental pIC50, ${y}_{pred}$ = predicted pIC50, and ${\bar{y}}_{pred}$ = mean of predicted pIC50.

## Supplementary information

Supplementary information.

Supplementary information2.

Supplementary information3.
