# Supplementary material for: Cheminformatics Analysis and Modeling with MacrolactoneDB
Source: Sci Rep. 2020 Apr 14;10:6284. doi: 10.1038/s41598-020-63192-4 (PMC7156526; doi:10.1038/s41598-020-63192-4)
Supplement: Supplementary file 1 — Supplementary information. [file 41598_2020_63192_MOESM1_ESM.pdf]

Supplemental material

Cheminformatics Analysis and Modeling with MacrolactoneDB

Phyo Phyo Kyaw Zin[1,2], Gavin Williams[1,3] and Sean Ekins[3,4*]

[1]Department of Chemistry, North Carolina State University, Raleigh, NC, USA.

[2]Bioinformatics Research Center, North Carolina State University, Raleigh, NC, USA.

[3]Comparative Medicine Institute, North Carolina State University, Raleigh, NC, USA.

[4]Collaborations Pharmaceuticals, Inc., 840 Main Campus Drive, Lab 3510, Raleigh, NC 27606, USA.

*To whom correspondence should be addressed: Sean Ekins, E-mail address: sean@collaborationspharma.com, Phone: +1 215-687-1320

Short running title: MacrolactoneDB

Keywords: Cheminformatics, Machine learning, Macrolactones, Molecular descriptors.

Supplemental Methods 1

MacrolactoneDB

The web interface of MacrolactoneDB (http://macrolact.collabchem.com/), provides options to subset macrolactones based on multiple criteria for which users can provide a range or a single minimal, maximal threshold value. For many filters, users can fill in both entries to provide a range wherein the first entry is designated for minimal values of the range and the later for the maximal. Providing only the first or the last entry designates a threshold value for minimal and maximal margins respectively. For example, if a numerical value "1" is provided in the first entry and the second entry is left blank for the filter e.g "Number of Sugars", the compounds with at least one sugar moiety will be filtered. If, however, "1' is filled only in the second entry and the first entry is left empty, the compounds with at most one sugar moiety will be selected. For structures with exactly one sugar, users can submit "1" in both the first and second entries for the "Number of Sugars" filter.

Below is a list of filters currently employed in MacrolactoneDB:

Ring Filters

1. Ring size: Ring size is defined as the number of atoms in the core ring structure with ring size larger than 12. MacrolactoneDB contains macrolactones which are at least 12-membered ring structures.
2. Number of rings: Some structures have several rings embedded. If users are looking to restrict the chemical space by number of rings, they can do so by inputting minimum and maximum values for the number of rings. ≥3 membered rings are accounted for.

3. Number of large rings (≥12-membered): Some macrolactones have two or more large ring structures attached together (large rings are ≥12 membered macrolactones). In this parameter, only large rings will be accounted for.
4. Number of fused rings
5. Number of aromatic rings

The filters on 4) the number of fused rings and 5) the number of aromatic rings are computed by Mordred descriptor calculator[1].

Macrolide Filters

6. Number of Sugars: Classic macrolides such as Erythromycin, Azithromycin, etc. have one or more glycosylated sugars attached to the core ring structures. If users intend to extract "macrolides" with any number of sugars, users can put "1" in the first entry and leave the second entry blank.
7. Number of esters in large rings (≥12-membered): Some of the macrolactones in MacrolactoneDB contain two or more esters within the core rings. Classic macrolides normally contain one ester within the core rings. Users can filter classic macrolides using this filter by submitting "1" in both the first and second entries.

Molecular Property Based Filters

8. Molecular weight: the mass of one mole of a given molecule; users can restrict the chemical space of macrolactones by their mass. It is useful for users who are interested in studying only small molecules (i.e. <900 Daltons) or larger macrolactones.

9. SlogP: hydrophobicity as assessed by the fragment-based octanol/water coefficient partition
10. Meet Lipinski Rules? Filter compounds on whether they are within druglike or oral bio-available region according to Lipinski's rule of five; backend module by Mordred descriptor calculator[1]. Users can select "Whatever" for no preference; which will provide a chemical dataset of compounds whether they are within the druglike region or not.

Bioactivity Filters

11. Activity Reported? Filter compounds by whether their bioactivities have been reported in ChEMBL. Users can select "Whatever" for no preference. "Whatever" option will provide a chemical dataset of compounds whether their bioactivities are reported or not.

Supplemental Methods 2

Computing Details for QSAR Modeling

All computing for RF, NB, SVR, KNN was done on 8 CPUs, 32GB RAM Intel Xeon W-2104 CPU, 64-bit operating system. All computing related with DNN calculations were done on Intel Xeon E5-2630 server running CentOS Linux 7 with 132 GB memory and four NVIDIA Corporation GP102 GPUs. DNN QSAR models were built with Keras 2.2.2 functional API, TensorFlow backend with GPU acceleration, NVIIA CuDNN libraries, CUDA 10.1; RF, NB, SVR, KNN were built with scikit-learn[12].

The scripts for data cleaning, manipulation, computing descriptors / fingerprints and developing QSAR models were written in python 3.6.3. The following modules were used: pandas[13], numpy[14], scipy[15], RDKit[8] and mordred[1]. SMILES for macrolactone ligands were standardized with molvs module[16] and used as input for descriptor / fingerprint generation.

Supplemental Methods 3

Machine learning Methods

Random Forest (RF) – an ensemble learning method that uses consensus of decision trees to predict the endpoint for regression problems[17,18]. RFs are robust to hyperparameter tuning and work well with different types of molecular descriptors. Known as the "gold standard", they have been shown to perform reasonably well for small and large chemical datasets.

Naïve Bayes (NB) – a probabilistic algorithm based on Bayes' theorem that assumes features are independent and distributed differently. Despite its simplicity, NB predictions are more accurate than other complex ML methods, especially for classification tasks. It is widely used in cheminformatics due to easy implementation and extremely fast computation.

Support Vector Regression (SVR) – a kernel regression function that finds a hyperplane or set of hyperplanes in a high-dimensional space. It is one of the most popular methods in cheminformatics commonly used to predict toxicity related endpoints[19–24].

K nearest neighbors (KNN) – one of the simplest algorithms which predicts a numerical target based on K closest or most similar neighbors[25]. KNN is widely used in a plethora of cheminformatics studies to predict physicochemical properties (melting point[26,27], boiling point[28], logP[27,28], aqueous solubility[27,29], etc.), drug clearance[30], mutagenic potency[31], odor characteristics of compounds[32,33], etc.

Deep Neural Nets (DNN) – one of the most popular methods in Cheminformatics, which have outperformed traditional MLs from time to time, especially when large training data is used. They are effective in tackling problems of any complexity level; from simple linear problems to

highly complex high-dimensional data. DNN can learn hidden patterns and relationships within the data without needing the domain experts to extract important features as in simpler traditional ML algorithms.

Consensus (CSS) takes the average prediction of 5 MLs (RF, NB, SVR, KNN and DNN).

Hybrid takes the average of two best performing ML methods. In our case studies, we applied two hybrids which are RF_KNN (average predictions from RF and KNN) and RF_DNN (average predictions from RF and DNN).

Supplemental Results 1

Cluster Analysis of Macrolactone Ligands

In the Hepatitis C dataset, we found an activity cliff (two 19-membered macrolactones indicated with red arrows in **Figure S6B**) by ECFP6, fragment-based approach. Their structures were shown in **Figure S6C**. The only difference between these two structures was a N2 (tertiary amine in the piperidine component, highlighted yellow in **Figure S6C**). Despite this minor structural difference, CHEMBL601195 and CHEMBL591648 afforded pIC50s of 7.22 and 5.84 respectively. They were however clustered relatively far apart and identified to be less similar via mordred_mrc. This demonstrates that despite high structural similarity, they had different molecular properties accounted for by mordred_mrc which contributed to various pIC50s and an activity cliff. Applying mordred_mrc to QSAR models may establish better chemical relationship between molecular properties and biological endpoints for these types of chemical analogues, while ECFP6 may have some challenges in this regard.

Additionally, we found a chemically unique macrolactone (CHEMBL2407590) based on mordred_mrc (Figure S6**A**, blue arrow), yet not as unique based on ECFP6 (**Figure S6B**, blue arrow). Its structure was shown in **Figure S6D**. It was a 33-membered peptide macrolactone, an attractive example to predict with our machine learning models to assess the relevance of structural fragment-based approaches like ECFP6 (10-fold CV predictions shown in **Figure S19B** and in **Results S4**).

In the T-Cells Dataset, an activity cliff was identified between CHEMBL422367 and CHEMBL347589 (blue cluster) based on ECFP6 (**Figure S7**). The former afforded experimental pIC50s of 8.67 and the later 5.88. With mordred_mrc, they were, however, located in different

clusters occupying similar shades of pIC50s. In other words, the activity cliff identified via ECFP6 was resolved in mordred_mrc. The structures were shown in **Figure S7D**. Herein, mordred_mrc captured the different molecular properties derived from the structural differences at C15 (highlighted yellow in **Figure S7D**). In this scenario, reducing the polarity of the side chain by replacing the ethanone component to a methyl significantly improved the activity of the analog.

Another structure which stood out in mordred_mrc based on relatively low pIC50 yet clustered with chemicals having higher pIC50s was shown in **Figure S7E**. It was however clustered together with other structures sharing similar pIC50s by mordred_mrc. It contained the same core scaffold as all the other chemical analogues in the previous purple and blue clusters with variations in the structural motif at C15.

Supplemental Results 2

Distribution Analysis of MRC Properties

We additionally looked at the distribution of ring sizes present in these case studies along with the distribution of pIC50 and number of compounds associated with corresponding ring sizes (**Figure S8**). 15-membered macrolactones (the most common ring size with 204 compounds) has a relatively lower variation in pIC50 values which range from 4.89 to 8.61. It might be ideal to model just 15-membered ring structures since the applicability domain would be limited and the performance of the model could be improved, however it could also introduce bias into our models. We can learn a lot from such large-scale modeling with all the machine learning algorithms and descriptor sets in this study. We do not know with certainty the level of influence of ring sizes or how critical they are towards their inhibition. Additionally, if implicit fingerprints like ECFP6 can sufficiently encode important features of these ring structures and predict biological endpoints, fragment-based descriptors may be an appropriate approach for building predictive models for structures with varying ring sizes, even for very large complex structures. Thus, restricting the dataset based on the most prevalent ring size would limit our exploration and understanding of this structural class. We used the entire dataset of pIC50 and the modeling results to better understand and gain insights into macrolactones. Additionally, due to the unbalanced distribution and limited variation of ring size (**Figure S8A**), sugars (**Figure S9**) and core esters (not reported; 221 macrolactones have 1 core ester and 2 macrolactones have 2 core esters) in the dataset of case study I (Plasmodium falciparum), we do not expect the prediction boost from adding mrc descriptors to mordred. We reported 10-fold CV predictions from our QSAR modeling workflow on CHEMBL2303629 and CHEMBL487366, two structures with highly

unique and complex ring sizes (the only 27-, 36- membered macrolactones) from the Plasmodium falciparum macrolactone ligand dataset.

In the second case study with Hepatitis C Virus, 18-membered macrolactones were the most dominant structures (65 compounds) with pIC50 ranging from 6.33 to 8.7. 19-membered macrolactones (12 compounds) covered a similar range of pIC50 from 6.22 to 8.07, whereas 20-membered macrolactones covered a larger pIC50 range from 4.5 to 8.01. From this dataset, it would be interesting to observe our machine learning predictions on ring size-based outliers such as 14, 21, and 33-membered macrolactones. Similar to the first case with Plasmodium falciparum, we do not expect to see the prediction boost from our mrc descriptors to mordred in the second case study with Hepatitis C due to limited variation and heavily uneven distribution in ring sizes (**Figure S8B**), sugars and core esters (not reported). In **Results S4**, we show model predictions on CHEMBL591604 (the only 21-membered) and CHEMBL2407590 (the only 33-membered macrolactones), the two largest ring structures from Hepatitis C macrolactone ligand dataset.

The T-cells target, our third case study, has the least variation in terms of ring sizes which account for 20, 21 and 29-membered macrolactones (**Figure S8C**), sugars and core esters (not reported). 21-membered ring size was the most dominant among all others with a total number of 87 compounds and covered larger pIC50 distribution which range from 4.93 to 9.74. Due to the small variation and uneven distribution of these properties, we do not expect to see a noticeable impact of mrc descriptors by complementing mordred descriptors.

Supplemental Results 3

Tuning parameters and feature extraction techniques to improve performance

For all targets, we implemented a) base ML models with no parameter tuning, b) models with more than 80% PCA feature extraction and tuned parameters, c) models with tuned parameters (**Figure S1**). For base models, only default parameters were used; no parameter tuning / selection was performed. For pca_tuned and tuned models, the best hyperparameters were selected by performing randomized search on 100 sample parameter settings with 10-fold cross-validations on the associated full datasets. The hyperparameters used in grid search areas across 5 MLs for pca_tuned and tuned models have been provided in **Table S4**. Additionally, DNN models use 300 training epochs with early stopping method to prevent overfitting. The hyperparameters for DNN covered in this study are provided in **Table S5**.

For all case studies of Plasmodium falciparum, Hepatitis C and T-cells datasets, we first generated heatmaps for eight machine learning (ML) models and six descriptors (excluding mrc descriptors) for base, pca_tuned and tuned models in the evaluation metrics of R^2 – coefficient of determination, and MAE – mean absolute error. All the modeling results in this study were consistently from 10-fold cross-validation. The results from mrc descriptor set were excluded due to consistently low performance in comparison to the rest; thereby affecting the heatmap. In our heatmaps, green is associated with “good” performance, while red is associated with “poor” performance; thus “green” indicates high R^2 and low MAE, and “red” indicates low R^2 and high MAE. The heatmaps of R^2 and MAE across base, pca_tuned and tuned models for Malaria, Hepatitis C, T-cells have been provided in **Figure S10**, **Figure** S11 and **Figure** S12 respectively. In

all three case studies, pca_tuned or tuned models had better performance than base models (greener in **Figure S10**, **Figure** S11, **Figure** S12) though it varies from one ML algorithm to another.

DNN and RF methods which can discriminate and select features on their own without needing much feature pruning or reduction work better with just parameter tuning. In fact, their performance worsened in all three cases when the PCA feature extraction technique was applied. It can be seen in the RF (**Figure S13A**) and DNN (**Figure S13B**) boxplot analysis of R^2 distribution across six descriptor sets for pca_tuned and tuned methods in three case studies. The SVR algorithm, which was observed to perform consistently well across the descriptor sets with the PCA-tuned method, performed worse in the tuned method in which ≥80% PCA feature extraction method was removed (**Figure S14**). In this scenario, applying PCA that explains 80% of the variables to the SVR algorithm boosted the performance because feature selection and/or extraction is an important part of developing good SVR models. It was perhaps due to removing less relevant features along with the noise that could be involved in 20% least explainable variables by PCA.

To interpret more easily across all case studies, we generated an average R^2 and MAE from 10-fold cross-validation of eight ML models and six descriptors (excluding mrc descriptors) for base, pca_tuned and tuned methods separately. It allowed us to compare the general performance of these three methods and see the impact of ≥80% PCA feature extraction and parameter optimizations. The resulting clustered columns for R^2 and MAE for all three targets have been provided in **Figure S15**. The tuned method yielded the best performance in terms of R^2 and MAE across the case studies. Except for the first case study of Plasmodium falciparum

(CHEMBL364), PCA-tuned models also performed better than base models which only use default parameters.

Supplemental Results 4

Performance of ML Workflow with Macrolactones

In this section, we took a few unique macrolactone ligand samples from each case study (Plasmodium falciparum, Hepatitis C and T-cells targets) and assessed the previously conducted 10-fold cross validation predictions from machine learning workflow on these individual compounds. We previously analyzed the distribution of ring sizes in each case study and sampled those with unique ring structures or complex, challenging structures based on circular dendrogram analysis. These are chemicals of interest because they stand out from the rest of the dataset due to either their ring size, structural complexity or molecular properties. The goal of this analysis is to assess the predictive power of QSAR models on these "misfit" or "unique" macrolactones even though the training data did not include many structures within their chemical proximity. This is because larger, more complex macrolactone rings are harder to organically synthesize, and are thus more difficult to test and retrieve experimental values. Consequently, they cover a sparse chemical space even though they are still of high research interest due to favorable biological activities reported. The importance of QSAR modeling could certainly be emphasized if good predictions can be made on such complex, uniquely ring-sized molecules especially if the training set barely covers the chemical scope of these structures.

In the case study I with Plasmodium falciparum, we picked two chemicals with unique ring sizes: CHEMBL2303629 (27-membered), CHEMBL487366 (36-membered). The predictions on CHEMBL2303629 (27-membered) and CHEMBL487366 (36-membered) are shown in **Figure**

S18. These predictions within 0.1 of experimental biological endpoints are bolded and will be referred from now on as "top" predictions. CHEMBL2303629 is, in fact, a highly complex structure with two large rings (26- and 27-membered) fused together with five 5-membered rings and three 6-membered rings. The only other structure containing two large structures in the Malaria dataset was CHEMBL1682585, which contained 12- and 15-membered rings. Regardless of whether that analogue, CHEMBL1682585, was part of the training data in the cross-validation process, the predictions from machine learning workflow on CHEMBL2303629 was quite impressive (**Figure S18A**). In fact, 8 combinations of ML and descriptors afforded "top" prediction values. Another compound of interest, CHEMBL487366, was the only 36-membered macrolactone in the Malaria dataset affording experimental pIC50 of 6.1 (**Figure S18B**). It is relatively less complex than the previous structure (CHEMBL2303629); nonetheless, the biggest macrolactone in the Plasmodium falciparum dataset with only two 31-membered macrolactones as the second biggest (**Figure S8A**). The machine learning workflow had highly successful predictions on this compound; 11 combinations of ML and descriptors afforded "top" predictions, which fell within 0.1 of its experimental pIC50.

It is important to note the high success rate of our models affording eight and eleven "top" predictions on these two uniquely ring-sized and complex molecules of CHEMBL2303629 (27-membered) and CHEMBL487366 (36-membered) respectively though the training set was dominated heavily by 15-membered ring sizes (**Results S2**, **Figure S8A**).

In the second case study with Hepatitis C, we picked out two macrolactones with 21- and 33-membered macrolactones which were unique ring size-wise. The predictions from machine learning workflow on 21-membered (CHEMBL591604) and 33-membered (CHEMBL2407590)

were shown in **Figure S19**. The former, the second largest ring structure in the Hepatitis C dataset, had experimental pEC50 of 8.08 (**Figure S19A**). There was only one “top” predicted pIC50 of 8.01 afforded by KNN algorithm and “all” descriptors. The second chemical, CHEMBL2407590 (33-membered) was sampled out in the circular dendrogram analysis as a unique chemical based on mordred_mrc. It was the single largest ring structure among the dataset of Hepatitis C Virus macrolactone ligands (**Figure S8B**). The machine learning workflow had more success with this compound in achieving six “top” predictions (**Figure S19B**). Overall in this second case study, the QSAR models still performed well and afforded “top” predictions on these two challenging and large ring size macrolactones: one ML and descriptor combination on the 21-membered CHEMBL591604 and six on 33-membered CHEMBL2407590.

In the third case study, there were three ring size variations in total, and no singled-out macrolactone with unique ring size (**Figure S8C**). Thus, we looked at “misfit” compounds from the activity cliffs and reported the findings in the **Results S4**. The important takeaways from a close look at “misfit” macrolactones from these studies was the models’ high predictability on uniquely different (or large) ring structures especially with mordred_mrc, ECFP6 and RF algorithm. Though they were one-of-a-kind in terms of ring size and structural complexity, our models afforded top predictions on these macrolactones as shown in the case studies of Malaria and Hepatitis C targets.

In the third case study with the T-cell dataset, we observed several activity cliffs by mordred_mrc (**Figure S7A**) and ECFP6 (**Figure S7B**). The first activity cliff (purple cluster containing three compounds - CHEMBL269732, CHEMBL351140, CHEMBL158921) was identified by mordred_mrc (**Figure S7A**). The activity cliff was however resolved by ECFP6 since they were

clustered apart with other analogues sharing similar shades of pIC50s (purple arrows in **Figure S7B**). Their structures were shown in **Figure S7C**. In that cluster, the compounds shared the same core scaffolds, but are differentiated in the side chains at C15 (highlight yellow) which contributed to widely varying pIC50s of 9.41, 8.3 and 5.68. Even though they were classified to be similar by mordred_mrc, ECFP6 distinguished them based on minor structural differences. These structural variations may play an important role in the interactions with the protein target though they are similar in terms of chemical properties. In this example, the shorter side chain at C15 afforded a higher, more favorable pIC50.

Another activity cliff was observed between two chemicals (CHEMBL162354 and CHEMBL352172) based on both mordred_mrc and ECFP6 (orange oval indicated with orange arrows). Their structures were shown in **Figure S20A** and **Figure S20B** along with experimental pIC50s and QSAR 10-fold CV predictions. These analogues were highly similar structure-wise and property-wise with the only difference being a single methyl extension at C15 position in the core ring structure. However, they afforded vastly different experimental pIC50s of 5.37 and 8.58. It is surprising that reducing one methyl from the side chain significantly improved pIC50s. Perhaps, this small structural change played an important role in accommodating the compound to fit well within the binding site of the target or it induced different poses or conformations of the ligand that favors interaction with the amino acids in the binding pocket. It would be useful to couple with docking studies in another project to further analyze these sets of analogues to better understand the contributions of such structural differences.

An activity cliff was found between CHEMBL162354 and CHEMBL352172 (highlighted blue oval) from circular dendrogram analysis based on both mordred_mrc and ECFP6. It is important

to note again that these two structure2 differ slightly by a single methyl at C6 of the core ring structure yet yielded moderately different experimental pIC50s. Understandably, the machine learning workflow did not perform as well on these analogues as in the previous case studies. No combination of ML and descriptor set afforded “top” predictions; thus, the single closest prediction value to the experimental pIC50 were underlined to assess its proximity of the best prediction value. CHEMBL162354 afforded experimental pIC50 of 5.37. A closer look at the circular dendrogram based on ECFP6 (**Figure S7B**, orange oval indicated with orange arrows) revealed that it was the only one among four compounds in that cluster of CHEMBL352172, CHEMBL162354, CHEMBL347139, CHEMBL351140 affording the lowest pIC50. Consequently, our models predicted very high pIC50s for this compound, especially ECFP6 across MLs (**Figure S20A**). None yielded a predicted value lower than pIC50 of 6; the closest prediction came from DNN with 2Drdkit which predicted pIC50 of 6.24 which was 0.87 apart from experimental pIC50.

However, the machine learning workflow had better success at predicting the other analogue CHEMBL352172 (**Figure S20B**) with an experimental pIC50 of 8.58 even though no combination afforded “top” predictions. Again, it is important to note that in the recently mentioned “orange oval and the closest cluster”, it had a similar range of pIC50s with other chemical analogues. Perhaps, that contributed to our models predicting closer values for this compound. The closest prediction came from DNN with “all” descriptors which yielded an pIC50 of 8.68 which was exactly 0.1 away from the experimental pIC50, on the borderline of “top” prediction. In fact, our workflow overwhelmingly predicted pIC50s above 7.0.

We also sampled another compound CHEMBL347657 with an experimental pIC50 of 5.78 (**Figure S20C**), based on cluster analysis of T-cells dataset with mordred_mrc in Cluster Analysis

of Macrolactone Ligands. It was clustered along with other analogues sharing very different shades of pIC50s in mordred_mrc. However, it was clustered with analogues sharing similar range of experimental pIC50 based on ECFP6. Thus, we initially assumed that ECFP6 may perhaps be better at predicting the bioactivity of CHEMBL347657 than mordred_mrc. However, our workflow did not afford any "top" prediction for this compound as well, not even with ECFP6. The closest prediction with pIC50 of 6.45 came from DNN with "all" descriptors, 0.67 higher than the actual value. It should be noted that it would be unreasonable to expect our models to make highly accurate predictions for these challenging and "misfit" macrolactones, especially with a small training dataset in the first place.

Interestingly in this case study with T-cells, DNN was able to generate the closet predictions either with "all" or 2Drdkit descriptors on these challenging macrolactones. In general, the workflow had the least success on these complex macrolactones with T-cell dataset among all three cases. Even for compounds from uneven clusters with various endpoints, DNN was doing relatively better than other ML algorithms including combined approaches such as CSS, RF_DNN and RF_KNN. It is hopeful that we can increase the predictive power of DNN models with more training data addressing these slight structural variations and associated biological endpoints. We expect including molecular dynamics-based fingerprints or descriptors will enhance the predictability of our models especially in the case study of T-cells with minor structural differences causing activity cliffs.

Supplemental Data 1.

Curated structure activity data – data supplied as a separate file.

Table S1. The number of chemicals filtered for 13 databases at each filtering stage; workflow developed using SMARTS and RDKit Molecule Substructure Filter in Knime Analytical Platform.

Database	# compounds	# processed	# RS >= 12	# Ester in ring RS >= 12
ChEMBL	1870461	1870461	33910	10205
BindingDB	1606505	1486509	25736	2574
ZINC15 – natural products	220952	220397	4644	1516
ZINC15 – biogenic	304595	304040	5335	1729
PubChem	895	890	388	384
AfroDB	954	954	8	1
AfroMalariaDB	265	265	2	1
BIOFACQUIM	423	423	15	15
NANPDB	6842	6842	335	264
NuBBe	2336	2336	19	18
StreptomeDB	3991	3991	792	563
Tipdb	8856	8856	184	73
unpd	216959	216959	10335	5509
Total	4244034	4122923	81703	22852

ZINC15 – natural products, ZINC15 – biogenic, ChEMBL, were downloaded directly from the hosted websites; whereas only structures tagged as “macrolides” were downloaded from PubChem since it contains an overwhelming number of 96 million structures.

Table S2. Descriptive Statistics on Molecular Properties of MacrolactoneDB: Molecular Weight (MW), PSA (Polar Surface Area), SlogP (Hydrophobicity), HBA (Hydrogen Bond Acceptors), HBD (Hydrogen Bond Donors), NRB (Number of Rotatable Bonds), and Ring Size (RS).

Properties	MW	PSA	SlogP	HBA	HBD	NRB	RS
Minimum	194.13	26.30	-18.45	2	0	0	12
Maximum	4429.72	1438.93	21.74	87	48	154	55
Mean	786.58	212.68	3.10	12.67	4.63	9.21	17.40
Median	761.47	181.16	3.33	12	3	8	15
Mode	867.29	252.75	2.33	7	3	8	14
Standard Deviation	338.63	139.27	2.65	6.36	4.88	7.98	5.99
25th Percentile	550.31	131.03	1.76	8	2	4	14
50th Percentile	761.47	181.16	3.33	12	3	8	15
75th Percentile	923.49	239.34	4.78	16	5	12	19
90th Percentile	1174.60	367.14	6.00	20	10	17	26
count	13721	13721	13721	13721	13721	13721	13721

Table S3. SMARTS for sugar patterns (for mrc descriptors)

Sugar Patterns	SMARTS
**1**	[OX2;$([r5]1@C@C@C(O)@C1),$([r6]1@C@C@C(O)@C(O)@C1)]
**2**	[OX2;$([r5]1@C(!@[OX2,NX3,SX2,FX1,ClX1,BrX1,IX1])@C@C@C1), $([r6]1@C(!@[OX2,NX3,SX2,FX1,ClX1,BrX1,IX1])@C@C@C@C1)]
**combi**	[OX2;$([r5]1@C(!@[OX2,NX3,SX2,FX1,ClX1,BrX1,IX1])@C@C(O)@C1), $([r6]1@C(!@[OX2,NX3,SX2,FX1,ClX1,BrX1,IX1])@C@C(O)@C(O)@C1)]
**2 reducing**	[OX2;$([r5]1@C(!@[OX2H1])@C@C@C1), $([r6]1@C(!@[OX2H1])@C@C@C@C1)]
**2 alpha**	[OX2;$([r5]1@[C@@](!@[OX2,NX3,SX2,FX1,ClX1,BrX1,IX1])@C@C@C1), $([r6]1@[C@@](!@[OX2,NX3,SX2,FX1,ClX1,BrX1,IX1])@C@C@C@C1)]
**2 beta**	[OX2;$([r5]1@[C@](!@[OX2,NX3,SX2,FX1,ClX1,BrX1,IX1])@C@C@C1), $([r6]1@[C@](!@[OX2,NX3,SX2,FX1,ClX1,BrX1,IX1])@C@C@C@C1)]

Table S4. Hyperparameters covered in grid search areas for pca_tuned and tuned models.

MLs	Grid Search Area
**RF**	# estimators = [200, 400, 600, 800, 1000, 1200, 1400, 1600, 1800, 2000]<br>Max features = ['auto', 'sqrt']<br>Max depth = [None, 10, 20, 30, 40, 50, 60, 70, 80, 90, 100, 110]<br>Min samples split = [2, 5, 10]<br>Min samples leaf = [1, 2, 4]<br>bootstrap = [True, False]
**NB**	# estimators = [200, 288, 377, 466, 555, 644, 733, 822, 911, 1000]<br>fit intercept = [True, False]
**SVR**	kernel = ['linear', 'poly', 'rbf', 'sigmoid']<br>shrinking = [True, False]<br>degree = [1,2,3]
**KNN**	# neighbors = [1, 3, 5, 7, 9, 11, 13, 15, 17, 20]<br>weights = ['distance', 'uniform']<br>algorithm = ['auto']<br>leaf size = [10, 20, 30, 40, 50, 60, 70, 80, 90, 100]
**DNN**	Parameters used in this study are V0 to V19 from Table S5.

Table S5. Summary of 30 Neural Network Architectures Tested for Internal Cross-Validation

Models	# hidden layers	Activation functions	Optimizer	Weight regularization	Dropout	Range of nodes in hidden layers
V0	9	ReLU	RMSprop	Yes	No	512 – 4
V1	7	ReLU	RMSprop	Yes	No	200 – 5
V2	4	ReLU	RMSprop	Yes	No	64 – 20
V3	2	ReLU	RMSprop	Yes	No	64 – 16
V4	3	ReLU	RMSprop	Yes	No	90 – 15
V5	10	ReLU, LeakyReLU	RMSprop	No	No	64 – 8
V6	3	ReLU, LeakyReLU	RMSprop	Yes	No	16 – 8
V7	2	ReLU	RMSprop	Yes	No	52 – 13
V8	3	tanh, linear, ReLU	RMSprop	Yes	No	70 – 21
V9	7	linear, LeakyReLU	RMSprop	No	No	128 – 16
V10	9	ReLU, linear	Adam	No	No	512 – 4
V11	7	ReLU, linear	Adam	Yes	No	200 – 5
V12	4	ReLU, linear	Adam	Yes	No	64 – 20
V13	2	ReLU	Adam	Yes	No	64 – 16
V14	3	ReLU	Adam	Yes	No	90 – 15
V15	10	linear, tanh, LeakyReLU	Adam	No	No	64 – 8
V16	3	tanh, LeakyReLU	Adam	Yes	No	16 – 8
V17	2	ReLU	Adam	Yes	No	52 – 13
V18	3	ReLU, elu	Adam	Yes	No	70 – 10
V19	5	Selu, LeakyReLU	RMSprop	Yes	No	128 – 16

Table S6. Maximally Reported Y-randomization Results from 10 iterations of 10-fold Cross-Validation for case study I - Malaria (CHEMBL364), pIC50

R^2	*NB*	*SVR*	*KNN*	*RF*	*RF_KNN*
2Drdkit	0.05	0.06	0.04	0.03	0.04
all	0.11	0.07	0	0.05	0.04
ecfp6	0.1	0.08	0.03	0.06	0.04
maccs	0.06	0.04	0.04	0.01	0.03
mordred	0.09	0.07	0.03	0.03	0.04
mordred_mrc	0.05	0.03	0.02	0.01	0.01

Table S7. Maximally Reported Y-randomization Results from 10 iterations of 10-fold Cross-Validation for case study II - Hepatitis C (CHEMBL379), pIC50/pEC50

R^2	*NB*	*SVR*	*KNN*	*RF*	*RF_KNN*
2Drdkit	0.12	0.1	0.02	0.01	0.02
all	0.13	0.08	0.04	0.03	0.05
ecfp6	0.1	0.09	0.03	0.06	0.06
maccs	0.12	0.11	0.1	0.13	0.12
mordred	0.14	0.1	0.02	0.03	0.02
mordred_mrc	0.11	0.08	0.05	0.04	0.06

Table S8. Maximally Reported Y-randomization Results from 10 iterations of 10-fold Cross-Validation for case study III – T-cells (CHEMBL614309), pIC50

R^2	*NB*	*SVR*	*KNN*	*RF*	*RF_KNN*
2Drdkit	0.17	0.14	0.09	0.14	0.11
all	0.17	0.08	0.02	0.01	0.01
ecfp6	0.1	0.1	0.12	0.15	0.18
maccs	0.14	0.14	0.05	0.03	0.04
mordred	0.15	0.06	0.08	0.07	0.09
mordred_mrc	0.16	0.12	0.07	0.14	0.15

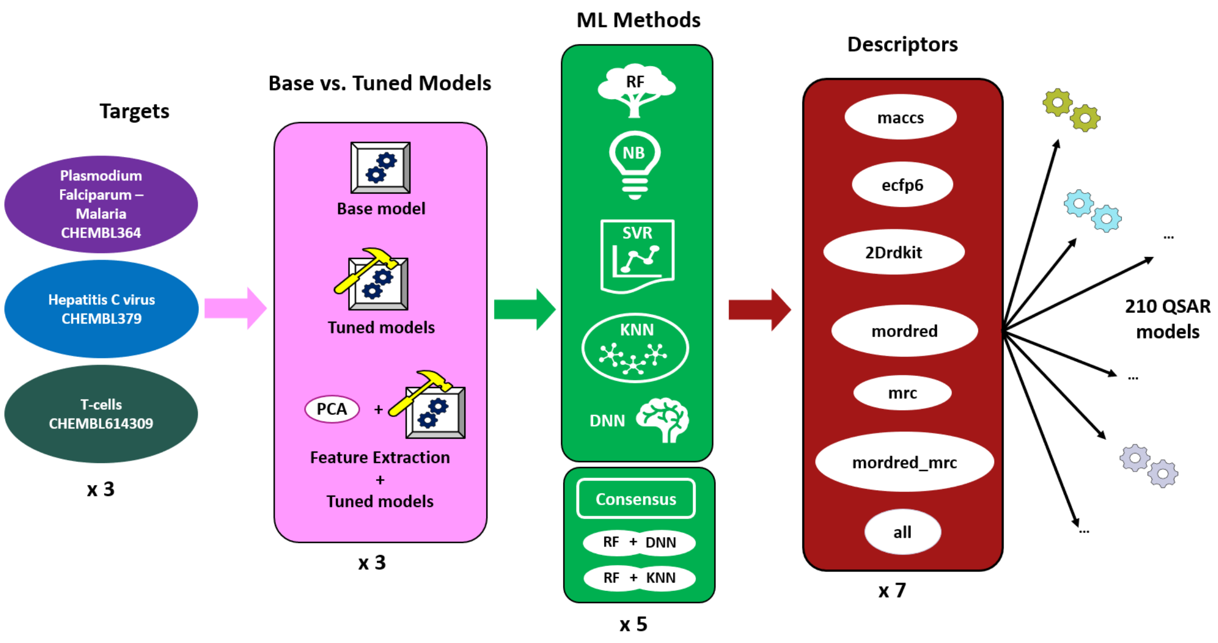

Figure S1. QSAR modeling workflow using 8 ML algorithms and 7 fingerprint / descriptor sets

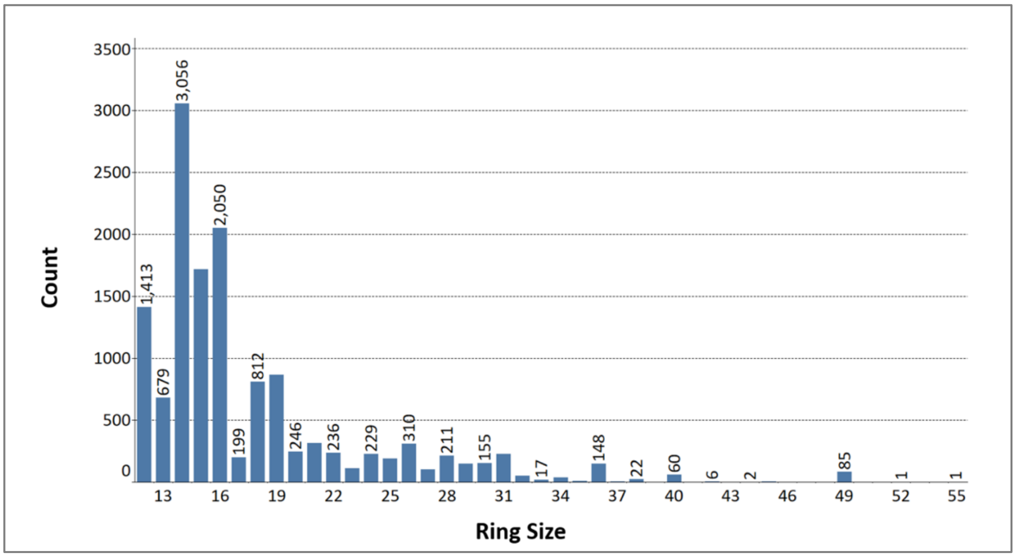

Figure S2. Distribution of Core Ring Sizes in MacrolactoneDB.

LIPINSKI	ACTIVE	INACTIVE
TRUE	91 (3%)	364 (13%)
FALSE	1134 (41%)	1162 (42%)

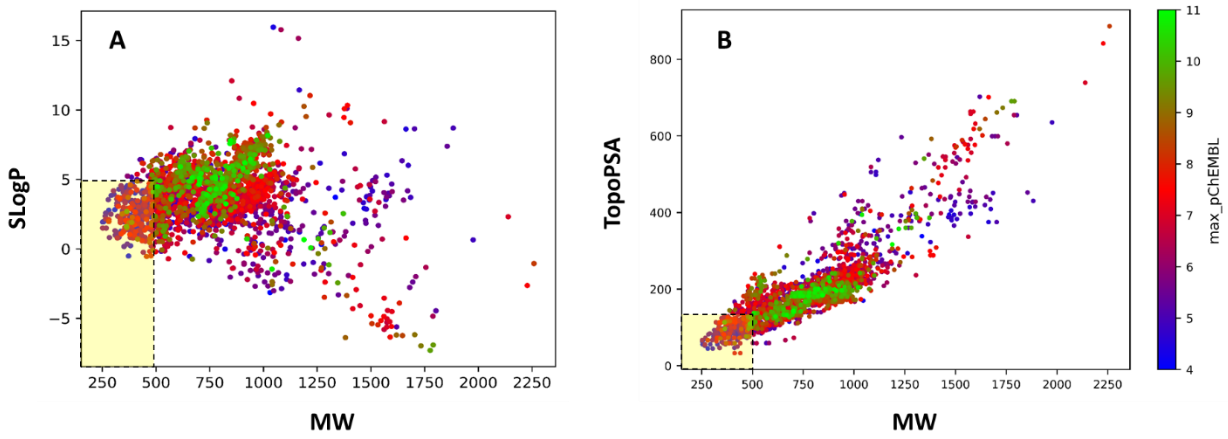

Figure S3. Statistics on the bioactivity of macrolactones and their agreement or lack thereof to Lipinski's rules. Scatter plots between (A) MW and SlogP, and (B) MW and TopoPSA. The compounds were colored based on their maximally reported pChEMBL values. Yellow regions indicate molecular properties within Lipinski's druglike rules.

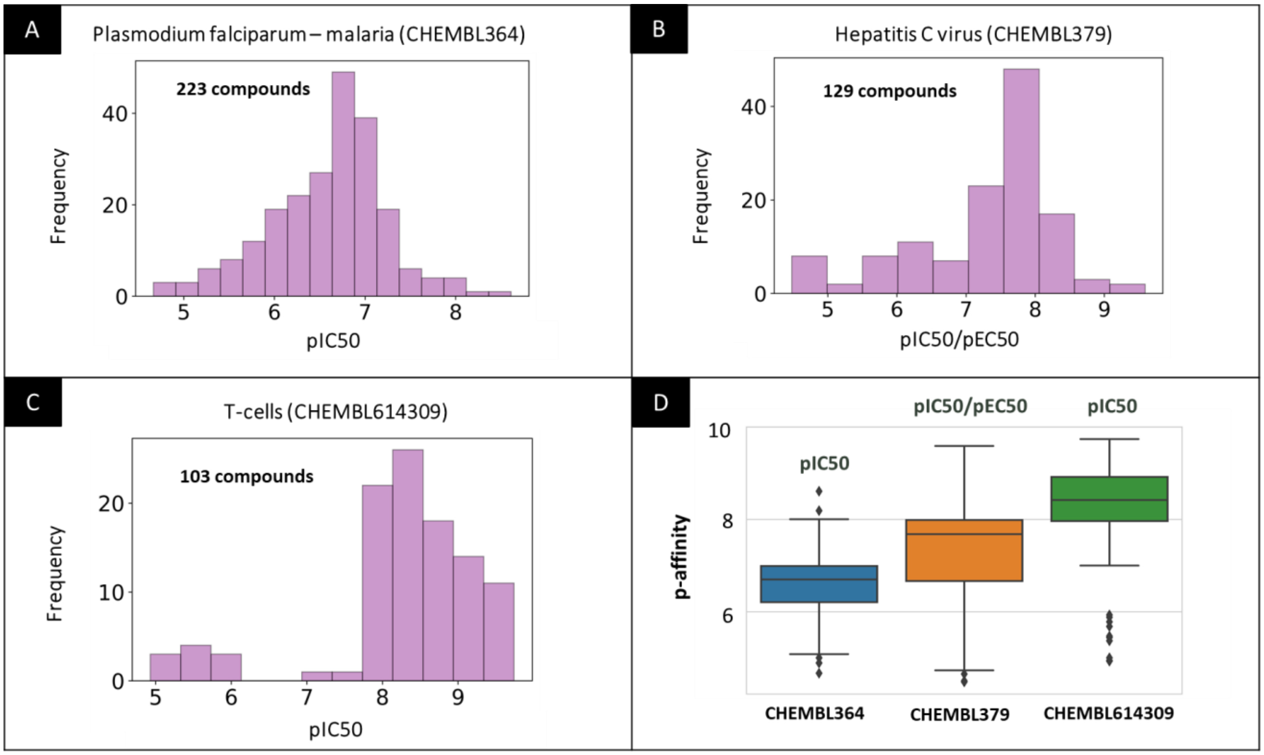

Figure S4. Distribution of (A) pIC50 values for Plasmodium falciparum (CHEMBL364), (B) pIC50/pEC50 values for Hepatitis C virus (CHEMBL379), (C) pIC50 values for T-cells (CHEMBL309), (D) Boxplot distribution of biological endpoints aka p-affinity for three case studies: Plasmodium falciparum (CHEMBL364), Hepatitis C (CHEMBL379) and T-cell (CHEMBL614309) targets.

Note: Outside this study, we attempted modeling with only pIC50s as endpoints for the Hepatitis C target; however, our models yielded poor performance as expected due to poor data distribution (not reported).

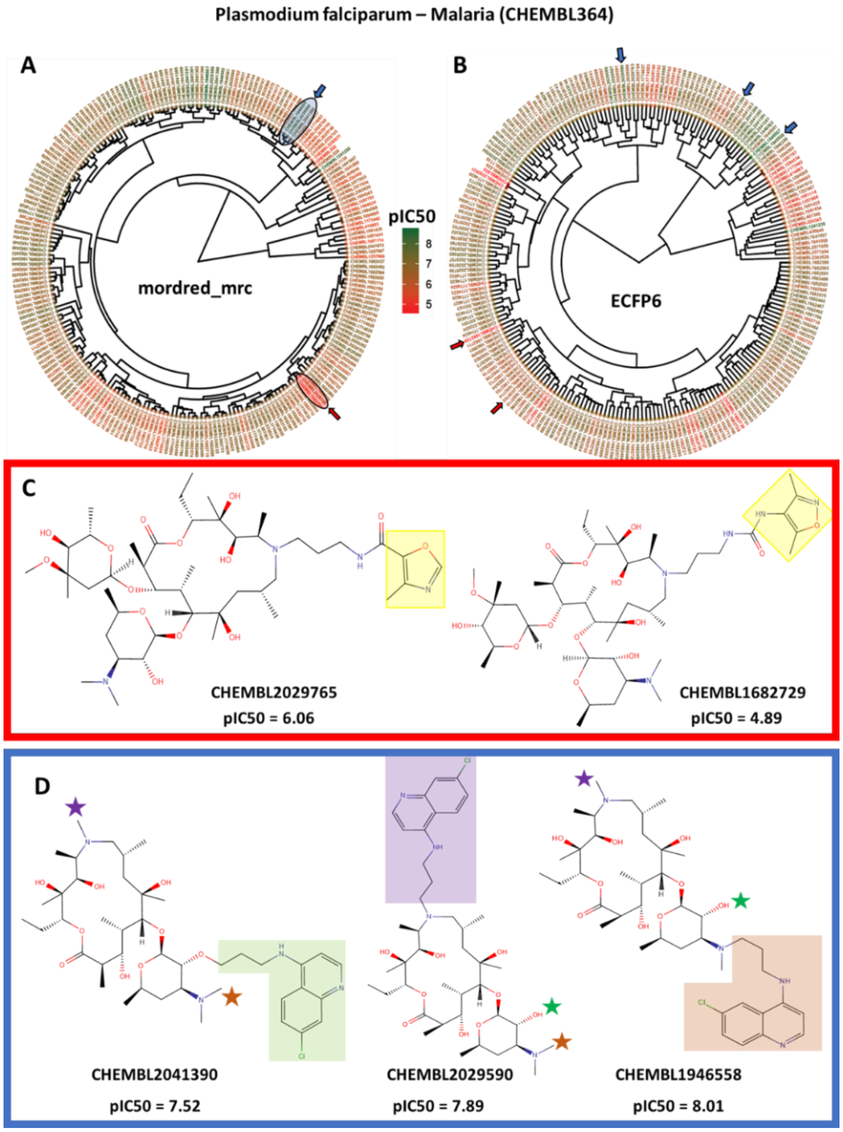

Figure S5. Circular dendrogram obtained from the hierarchical clustering of Plasmodium falciparum (CHEMBL364) dataset with Ward linkage, and Euclidian distance using (A) modred_mrc descriptors, (B) ECFP6 fingerprints. Compound nodes and names are colored according to their pIC50 value. Structures of (C) two chemicals indicated with red arrows in both mordred and ECFP6 dendrograms, and (D) three chemicals from the blue oval based on mordred and in different clusters indicated by blue arrows based on ECFP6. Colored star symbols indicate shared structural fragments among the chemicals.

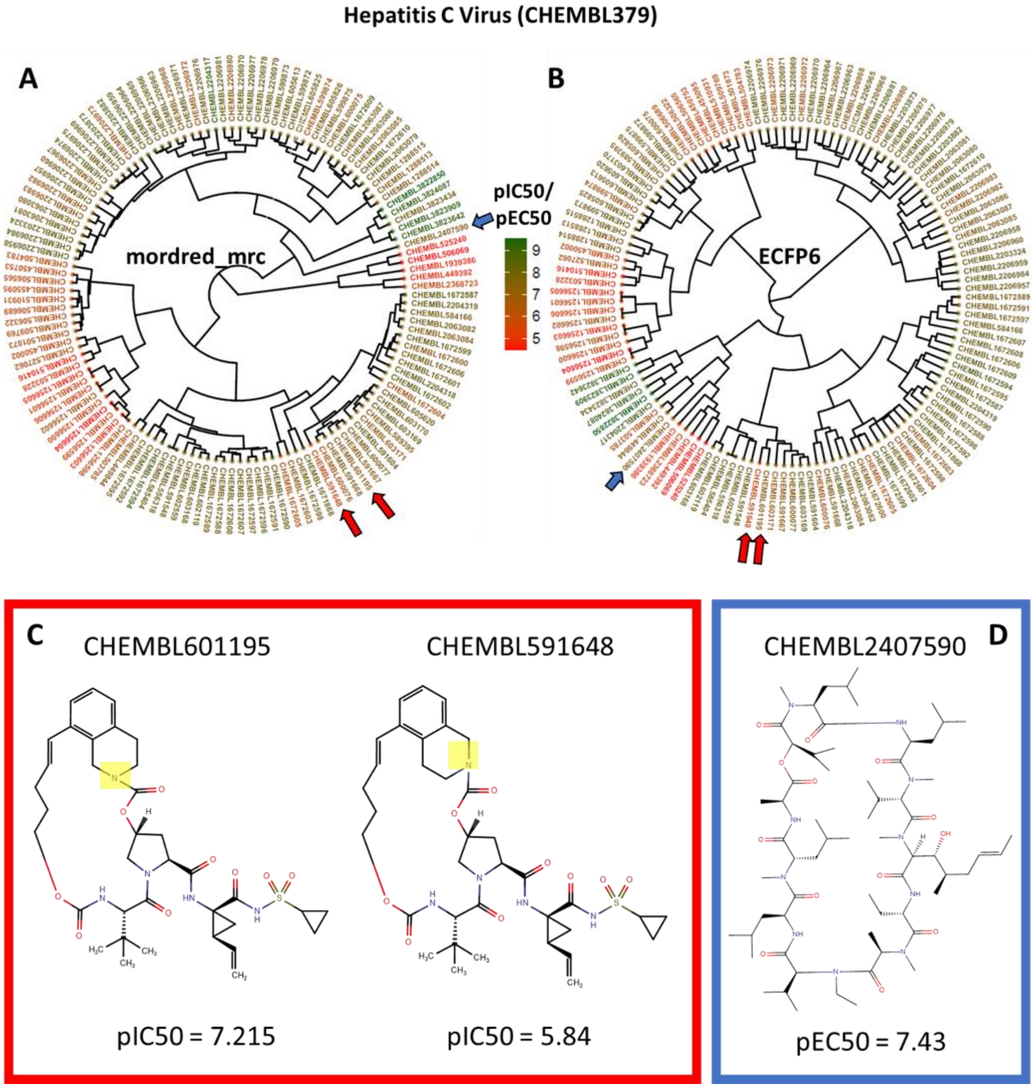

Figure S6. Circular dendrogram obtained from the hierarchical clustering of Hepatitis C virus (CHEMBL379) dataset with Ward linkage, and Euclidian distance using (A) modred_mrc descriptors, and (B) ECFP6 fingerprints. Compound nodes and names are colored according to their pIC50 value. Stuctures with activity cliff based on ECFP6 clustering are shown in (C). The structure of the most chemically unique macrolactone based on mordred_mrc dendrogram is shown in (D).

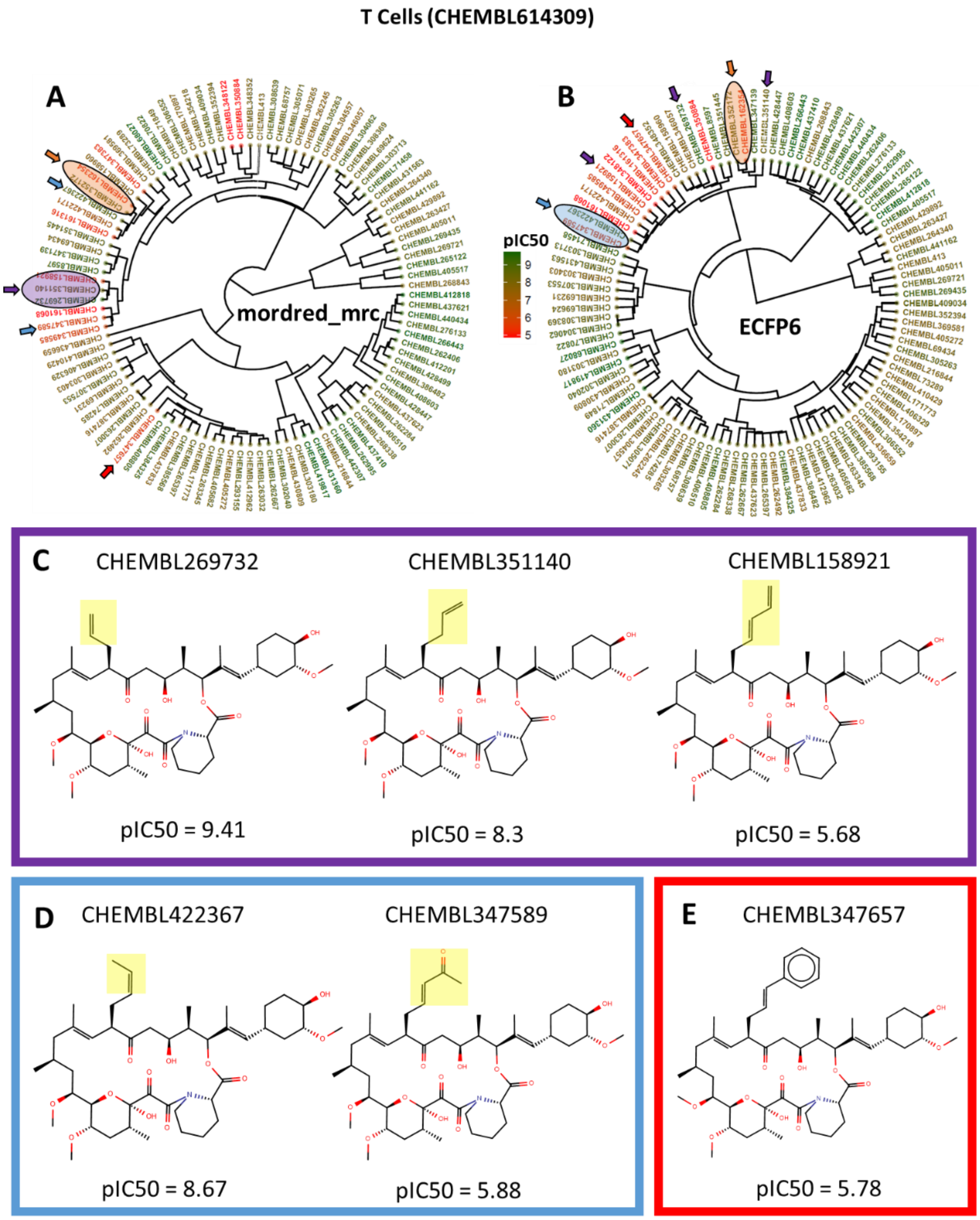

Figure S7. Circular dendrogram obtained from the hierarchical clustering of T-cells (CHEMBL614309) dataset with Ward linkage, and Euclidian distance using (A) modred_mrc descriptors, and (B) ECFP6 fingerprints. Compound nodes and names are colored according to their pIC50 value. Structures from purple oval or indicated by purple arrows are shown in (C), blue oval or indicated with blue arrows are shown in (D).

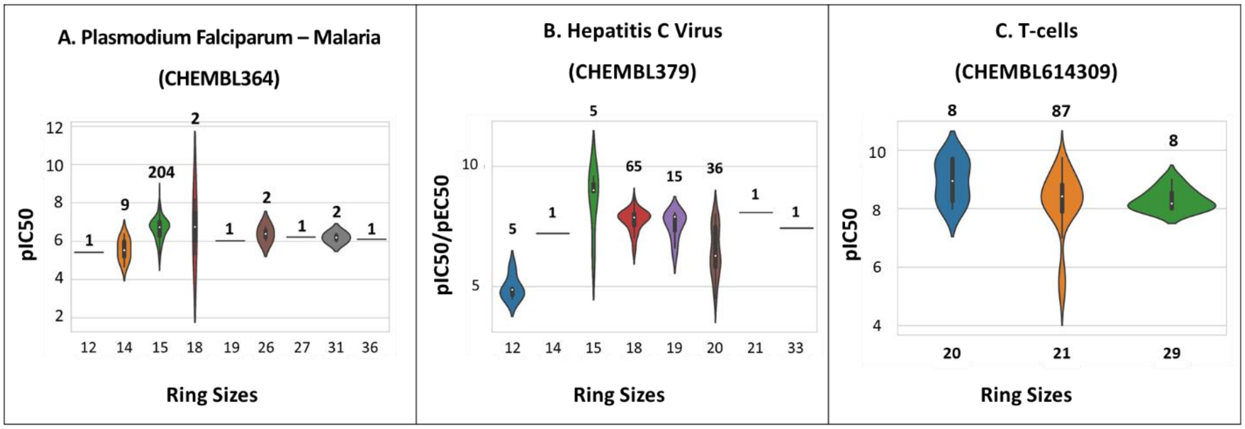

Figure S8. Violin plot analysis of ring size scaffolds with associated pIC50 and pChEMBL distributions for (A) Plasmodium falciparum (CHEMBL364), (B) Hepatitis C Virus (CHEMBL379), and (C) T-cells (CHEMBL614309) respectively. The number of compounds for associated ring sizes are reported above the corresponding violin shape distribution for each ring size.

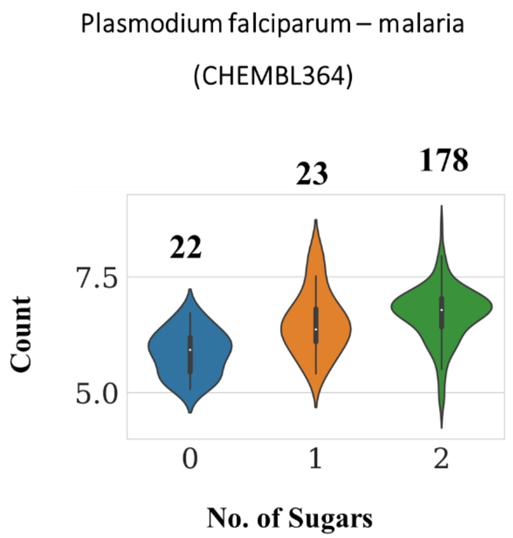

Figure S9. Violin plot analysis of sugars with associated pIC50 score distributions for Plasmodium falciparum (CHEMBL364). The number of compounds for associated ring sizes are reported above the violin shapes.

Case study I - Malaria (CHEMBL364): 10-fold Cross-Validation (pIC50)

A. Evaluation by R^2 (Coefficient of Determination)

R^2	NB	SVR	KNN	RF	DNN	CSS	RF_ DNN	RF_ KNN	n=223
mordred	0.53	0.36	0.51	0.57	0.56	0.61	0.58	0.63	base
mordred_mrc	0.52	0.35	0.52	0.56	0.52	0.61	0.57	0.61	
2Drdkit	0.44	0.37	0.51	0.55	0.45	0.57	0.57	0.57	
ecfp6	0.53	0.27	0.4	0.43	0.47	0.53	0.45	0.52	
maccs	0.35	0.32	0.32	0.38	0.39	0.43	0.38	0.43	
all	0.59	0.34	0.47	0.56	0.55	0.61	0.63	0.57	
mordred	0.35	0.52	0.58	0.56	0.44	0.58	0.58	0.54	pca_tuned
mordred_mrc	0.35	0.52	0.58	0.54	0.45	0.57	0.57	0.53	
2Drdkit	0.39	0.48	0.56	0.52	0.48	0.58	0.57	0.56	
ecfp6	0.42	0.51	0.44	0.43	0.37	0.49	0.46	0.43	
maccs	0.34	0.39	0.35	0.35	0.33	0.41	0.38	0.36	
all	0.41	0.51	0.48	0.47	0.48	0.52	0.5	0.49	
mordred	0.53	0.51	0.56	0.59	0.56	0.63	0.6	0.61	tuned
mordred_mrc	0.52	0.48	0.56	0.59	0.51	0.6	0.6	0.59	
2Drdkit	0.44	0.44	0.56	0.62	0.52	0.6	0.61	0.61	
ecfp6	0.53	0.52	0.43	0.5	0.57	0.55	0.48	0.56	
maccs	0.38	0.32	0.37	0.43	0.32	0.44	0.42	0.4	
all	0.59	0.59	0.47	0.59	0.6	0.63	0.64	0.57	

B. Evaluation by MAE (Mean Absolute Error)

MAE	NB	SVR	KNN	RF	DNN	CSS	RF_ DNN	RF_ KNN	n=223
mordred	0.32	0.39	0.34	0.31	0.33	0.3	0.32	0.29	base
mordred_mrc	0.32	0.39	0.33	0.32	0.34	0.31	0.32	0.3	
2Drdkit	0.36	0.38	0.34	0.33	0.36	0.31	0.32	0.31	
ecfp6	0.31	0.41	0.39	0.36	0.37	0.31	0.35	0.32	
maccs	0.38	0.4	0.4	0.38	0.4	0.36	0.38	0.37	
all	0.28	0.4	0.35	0.32	0.32	0.29	0.28	0.31	
mordred	0.38	0.33	0.32	0.31	0.37	0.31	0.31	0.32	pca_tuned
mordred_mrc	0.38	0.33	0.32	0.31	0.36	0.31	0.31	0.31	
2Drdkit	0.37	0.34	0.33	0.32	0.36	0.31	0.32	0.31	
ecfp6	0.36	0.33	0.37	0.37	0.39	0.34	0.36	0.35	
maccs	0.39	0.37	0.4	0.39	0.42	0.37	0.38	0.39	
all	0.36	0.33	0.35	0.35	0.35	0.33	0.33	0.34	
mordred	0.32	0.34	0.32	0.31	0.33	0.29	0.31	0.3	tuned
mordred_mrc	0.32	0.35	0.32	0.31	0.36	0.3	0.31	0.3	
2Drdkit	0.36	0.36	0.33	0.29	0.35	0.31	0.3	0.3	
ecfp6	0.31	0.32	0.37	0.33	0.3	0.3	0.34	0.3	
maccs	0.39	0.4	0.39	0.36	0.41	0.36	0.37	0.37	
all	0.28	0.3	0.35	0.31	0.28	0.28	0.27	0.32	

Figure S10. 10-fold Cross-Validation across eight machine learning algorithms and six fingerprint / descriptor sets for case study I with Plasmodium falciparum (CHEMBL364): (A) Coefficient of Determination (R^2), and (B) Mean Absolute Error (MAE).

Case study II - Hepatitis C (CHEMBL379): 10-fold Cross-Validation (pIC50/EC50)

A. Evaluation by R^2 (Coefficient of Determination)

MLs	NB	SVR	KNN	RF	DNN	CSS	RF_ DNN	RF_ KNN	n=129
mordred	0.68	0.59	0.64	0.61	0.56	0.7	0.68	0.65	base
mordred_mrc	0.68	0.58	0.65	0.66	0.51	0.69	0.7	0.64	
2Drdkit	0.65	0.5	0.69	0.66	0.61	0.7	0.71	0.67	
ecfp6	0.72	0.51	0.7	0.61	0.57	0.69	0.68	0.62	
maccs	0.67	0.52	0.68	0.68	0.58	0.7	0.72	0.67	
all	0.73	0.55	0.68	0.67	0.64	0.73	0.7	0.72	
mordred	0.64	0.67	0.7	0.67	0.65	0.73	0.7	0.71	pca_tuned
mordred_mrc	0.65	0.67	0.7	0.68	0.61	0.73	0.71	0.69	
2Drdkit	0.62	0.65	0.72	0.68	0.59	0.71	0.71	0.67	
ecfp6	0.71	0.71	0.69	0.73	0.57	0.72	0.73	0.67	
maccs	0.65	0.76	0.71	0.7	0.68	0.75	0.72	0.71	
all	0.74	0.74	0.73	0.72	0.6	0.75	0.7	0.74	
mordred	0.68	0.56	0.73	0.73	0.71	0.72	0.75	0.75	tuned
mordred_mrc	0.68	0.58	0.74	0.73	0.71	0.75	0.75	0.74	
2Drdkit	0.65	0.64	0.73	0.74	0.65	0.73	0.75	0.73	
ecfp6	0.72	0.64	0.7	0.74	0.68	0.72	0.73	0.72	
maccs	0.67	0.67	0.72	0.74	0.57	0.71	0.74	0.68	
all	0.73	0.66	0.73	0.73	0.65	0.73	0.71	0.76	

B. Evaluation by MAE (Mean Absolute Error)

MLs	NB	SVR	KNN	RF	DNN	CSS	RF_ DNN	RF_ KNN	n=129
mordred	0.42	0.59	0.44	0.45	0.51	0.42	0.42	0.45	base
mordred_mrc	0.42	0.6	0.43	0.41	0.53	0.42	0.4	0.44	
2Drdkit	0.46	0.59	0.42	0.43	0.5	0.43	0.41	0.44	
ecfp6	0.41	0.57	0.41	0.45	0.49	0.41	0.41	0.44	
maccs	0.44	0.48	0.42	0.43	0.51	0.41	0.4	0.43	
all	0.39	0.56	0.41	0.4	0.45	0.39	0.39	0.38	
mordred	0.47	0.43	0.42	0.43	0.48	0.4	0.41	0.43	pca_tuned
mordred_mrc	0.47	0.43	0.42	0.43	0.49	0.4	0.41	0.43	
2Drdkit	0.49	0.46	0.42	0.44	0.48	0.41	0.41	0.43	
ecfp6	0.41	0.4	0.44	0.4	0.5	0.41	0.42	0.44	
maccs	0.45	0.37	0.42	0.43	0.44	0.39	0.42	0.41	
all	0.39	0.37	0.39	0.4	0.48	0.37	0.4	0.38	
mordred	0.42	0.52	0.38	0.41	0.44	0.4	0.38	0.4	tuned
mordred_mrc	0.42	0.6	0.38	0.41	0.41	0.4	0.38	0.39	
2Drdkit	0.46	0.46	0.4	0.41	0.45	0.4	0.39	0.4	
ecfp6	0.41	0.48	0.4	0.39	0.44	0.4	0.39	0.39	
maccs	0.44	0.44	0.4	0.39	0.51	0.4	0.39	0.42	
all	0.39	0.44	0.4	0.41	0.46	0.39	0.4	0.37	

Figure S11. 10-fold Cross-Validation across eight machine learning algorithms and six fingerprint / descriptor sets for case study II with Hepatitis C Virus (CHEMBL379): (A) Coefficient of Determination (R^2), and (B) Mean Absolute Error (MAE).

Case study III - T-cells (CHEMBL614309): 10-fold Cross-Validation (pIC50)

A. Evaluation by R^2 (Coefficient of Determination)

MLs	NB	SVR	KNN	RF	DNN	CSS	RF_DNN	RF_KNN	n=103
mordred	0.53	0.22	0.44	0.51	0.5	0.62	0.62	0.56	base
mordred_mrc	0.54	0.22	0.44	0.66	0.57	0.68	0.71	0.62	
2Drdkit	0.34	0.22	0.41	0.45	0.47	0.53	0.55	0.5	
ecfp6	0.49	0.17	0.45	0.49	0.41	0.52	0.51	0.49	
maccs	0.17	0.17	0.18	0.12	0.04	0.14	0.07	0.16	
all	0.56	0.22	0.6	0.64	0.53	0.67	0.68	0.7	
mordred	0.26	0.58	0.56	0.54	0.54	0.65	0.6	0.62	pca_tuned
mordred_mrc	0.26	0.58	0.56	0.57	0.67	0.69	0.68	0.63	
2Drdkit	0.18	0.24	0.4	0.59	0.57	0.55	0.64	0.53	
ecfp6	0.44	0.51	0.48	0.42	0.41	0.53	0.47	0.47	
maccs	0.17	0.19	0.14	0.1	0	0.15	0.07	0.13	
all	0.56	0.65	0.56	0.62	0.59	0.68	0.65	0.64	
mordred	0.53	0.44	0.48	0.78	0.6	0.68	0.72	0.67	tuned
mordred_mrc	0.54	0.45	0.48	0.78	0.65	0.68	0.75	0.67	
2Drdkit	0.34	0.28	0.48	0.67	0.68	0.61	0.72	0.59	
ecfp6	0.49	0.5	0.48	0.53	0.55	0.55	0.56	0.52	
maccs	0.17	0.17	0.17	0.17	0	0.17	0.13	0.17	
all	0.56	0.57	0.6	0.78	0.62	0.7	0.74	0.73	

B. Evaluation by MAE (Mean Absolute Error)

MLs	NB	SVR	KNN	RF	DNN	CSS	RF_DNN	RF_KNN	n=103
mordred	0.55	0.64	0.58	0.51	0.61	0.49	0.5	0.51	base
mordred_mrc	0.55	0.64	0.58	0.45	0.58	0.47	0.43	0.48	
2Drdkit	0.62	0.61	0.58	0.54	0.66	0.53	0.54	0.52	
ecfp6	0.52	0.66	0.51	0.51	0.6	0.51	0.52	0.49	
maccs	0.64	0.62	0.7	0.74	0.97	0.7	0.84	0.7	
all	0.51	0.64	0.49	0.45	0.56	0.47	0.46	0.44	
mordred	0.65	0.52	0.52	0.51	0.6	0.46	0.48	0.47	pca_tuned
mordred_mrc	0.65	0.52	0.52	0.51	0.48	0.45	0.42	0.48	
2Drdkit	0.64	0.58	0.6	0.49	0.57	0.51	0.46	0.53	
ecfp6	0.54	0.52	0.53	0.56	0.63	0.49	0.51	0.53	
maccs	0.65	0.63	0.66	0.7	0.73	0.65	0.69	0.67	
all	0.52	0.47	0.5	0.49	0.53	0.43	0.44	0.47	
mordred	0.55	0.64	0.56	0.39	0.46	0.44	0.4	0.45	tuned
mordred_mrc	0.55	0.64	0.56	0.39	0.46	0.45	0.4	0.45	
2Drdkit	0.62	0.64	0.55	0.45	0.46	0.49	0.41	0.49	
ecfp6	0.52	0.53	0.51	0.49	0.49	0.49	0.47	0.49	
maccs	0.64	0.62	0.69	0.65	0.73	0.64	0.66	0.67	
all	0.51	0.52	0.49	0.39	0.47	0.42	0.4	0.42	

Figure S12. 10-fold Cross-Validation across eight machine learning algorithms and six fingerprint / descriptor sets for case study III with T-cells (CHEMBL614309): (A) Coefficient of Determination (R^2), and (B) Mean Absolute Error (MAE).

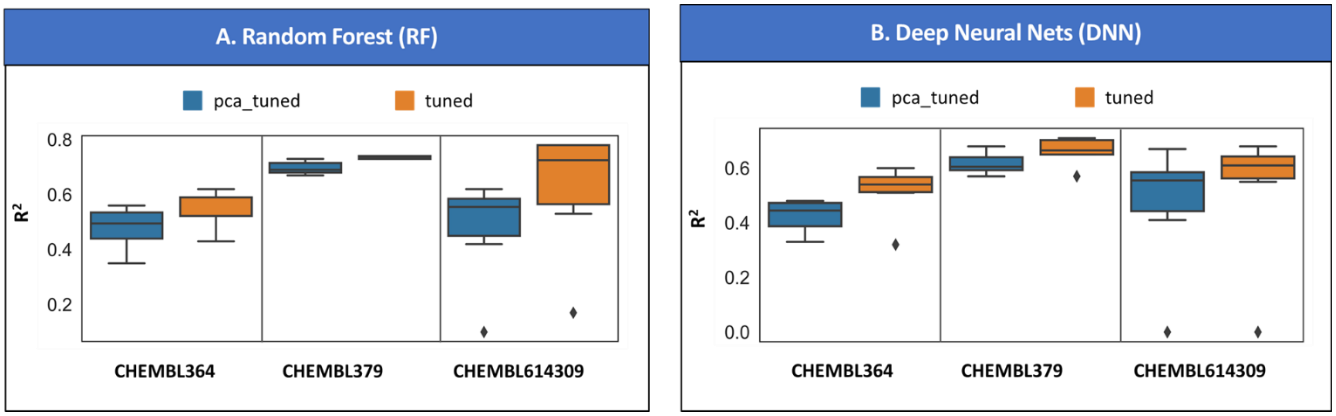

Figure S13. Boxplot comparison of pca_tuned and tuned methods for six descriptor sets across three case studies of Plasmodium falciparum (CHEMBL364), Hepatitis C Virus (CHEMBL379) and T-cells (CHEMBL614309) in terms of R^2 – coefficient of determination: (A) Random Forest (RF), and (B) Deep Neural Nets (DNN).

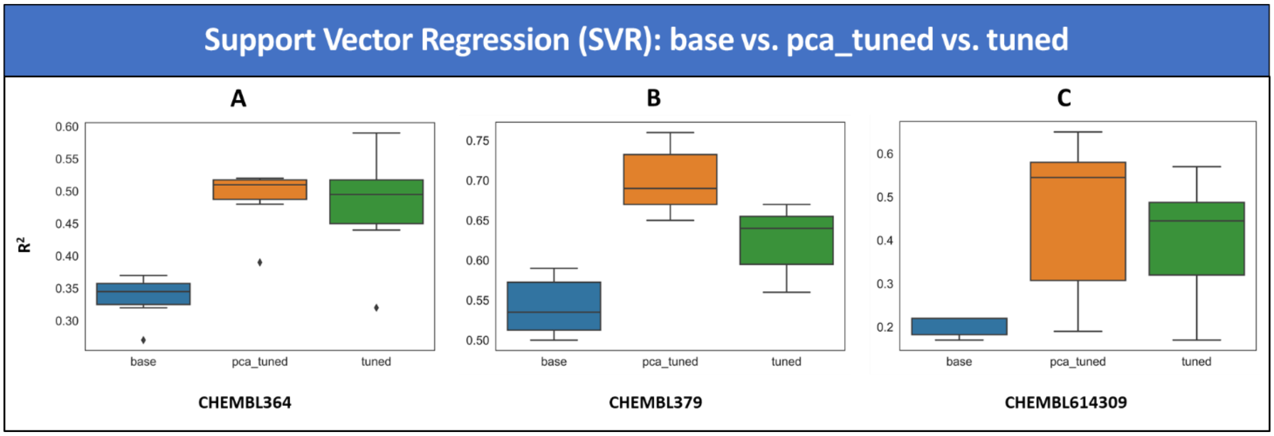

Figure S14. Distribution of Coefficient of Determination R^2 using Support Vector Regression (SVR) from six descriptor sets for base, pca_tuned and tuned methods for (A) Plasmodium falciparum (CHEMBL364), (B) Hepatitis C Virus (CHEMBL379), and (C) T-cells (CHEMBL614309).

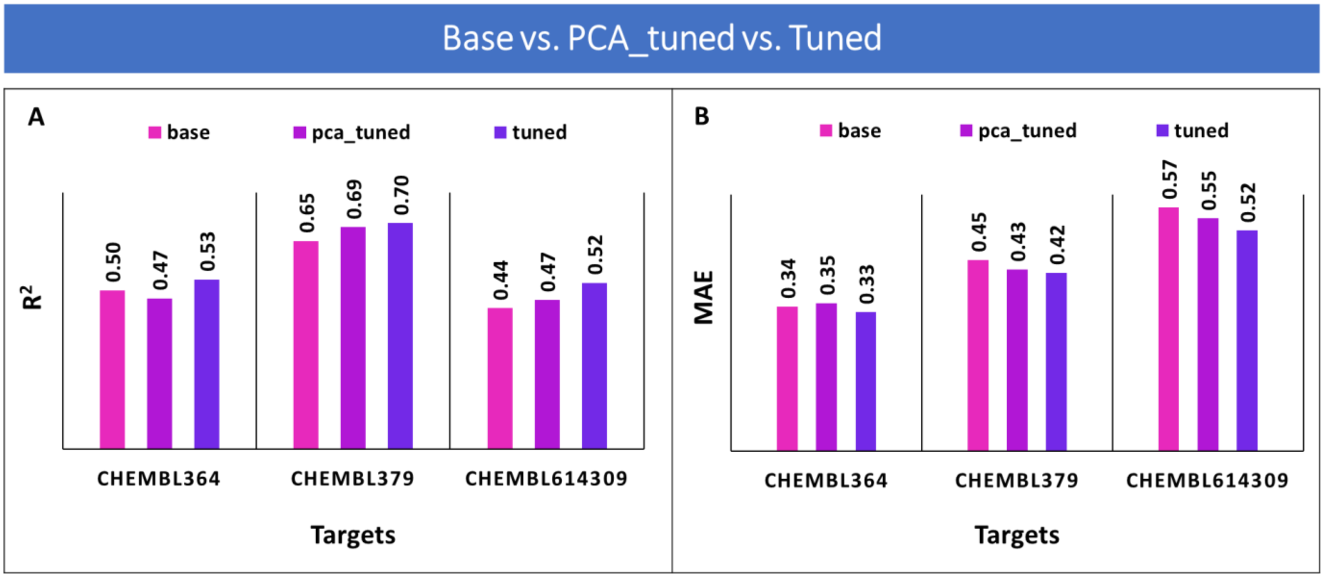

Figure S15. Comparison of base, pca_tuned, tuned methods from eight MLs and six descriptors (excluding mrc descriptors) used in the study across three case studies of Plasmodium falciparum (CHEMBL364), Hepatitis C Virus (CHEMBL379), T-cells (CHEMBL614309) for (A) coefficient of determination (R^2) and (B) mean absolute error (MAE).

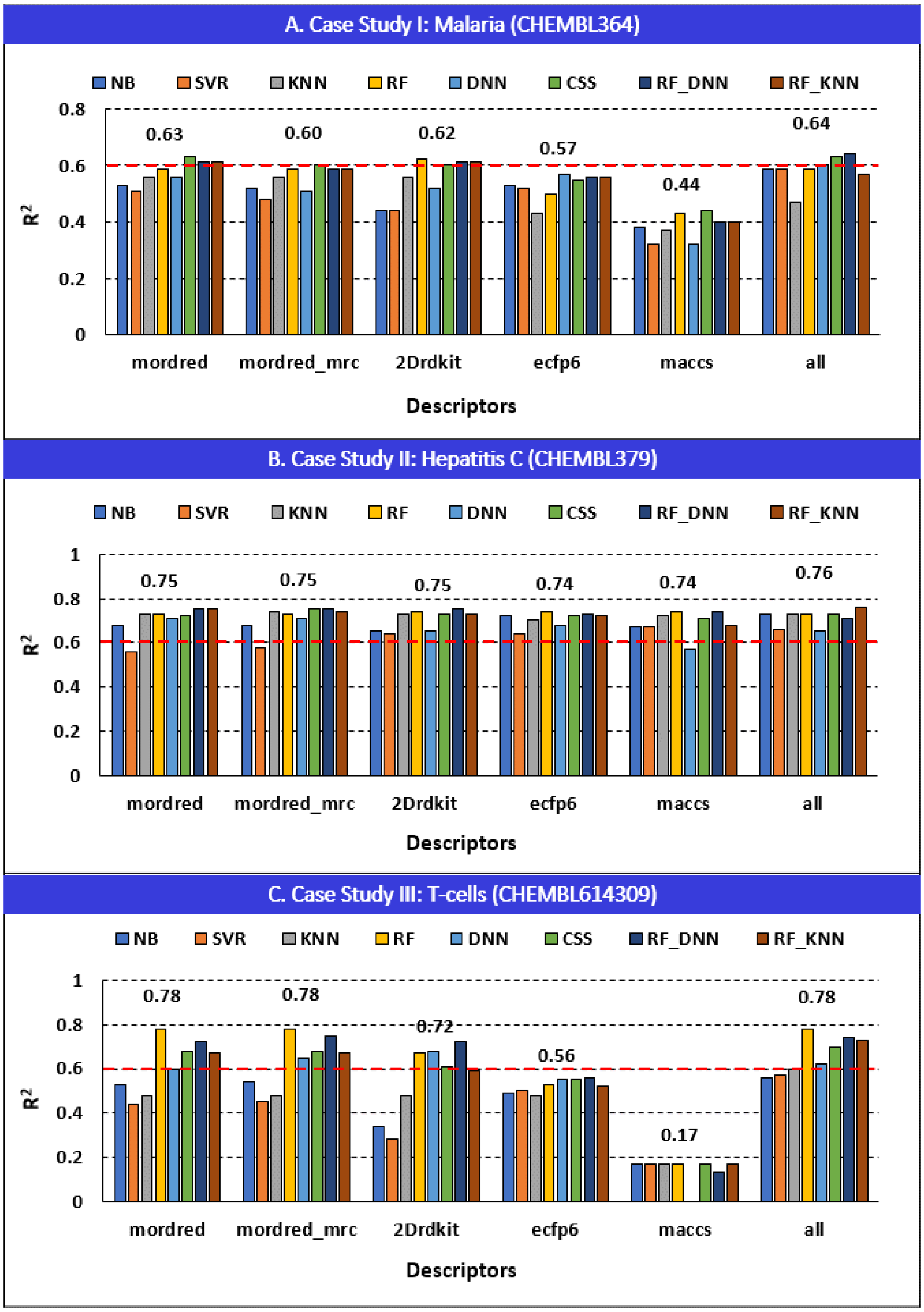

Figure S16. Clustered columns analysis of coefficient of determination (R2) from 10-fold cross validation for (A) Plasmodium falciparum (CHEMBL364), (B) Hepatitis C Virus (CHEMBL379), and (C) T-cells (CHEMBL614309). The numbers indicated on each set of clustered columns are the highest R^2 value from the associated set of 8 machine learning algorithms.

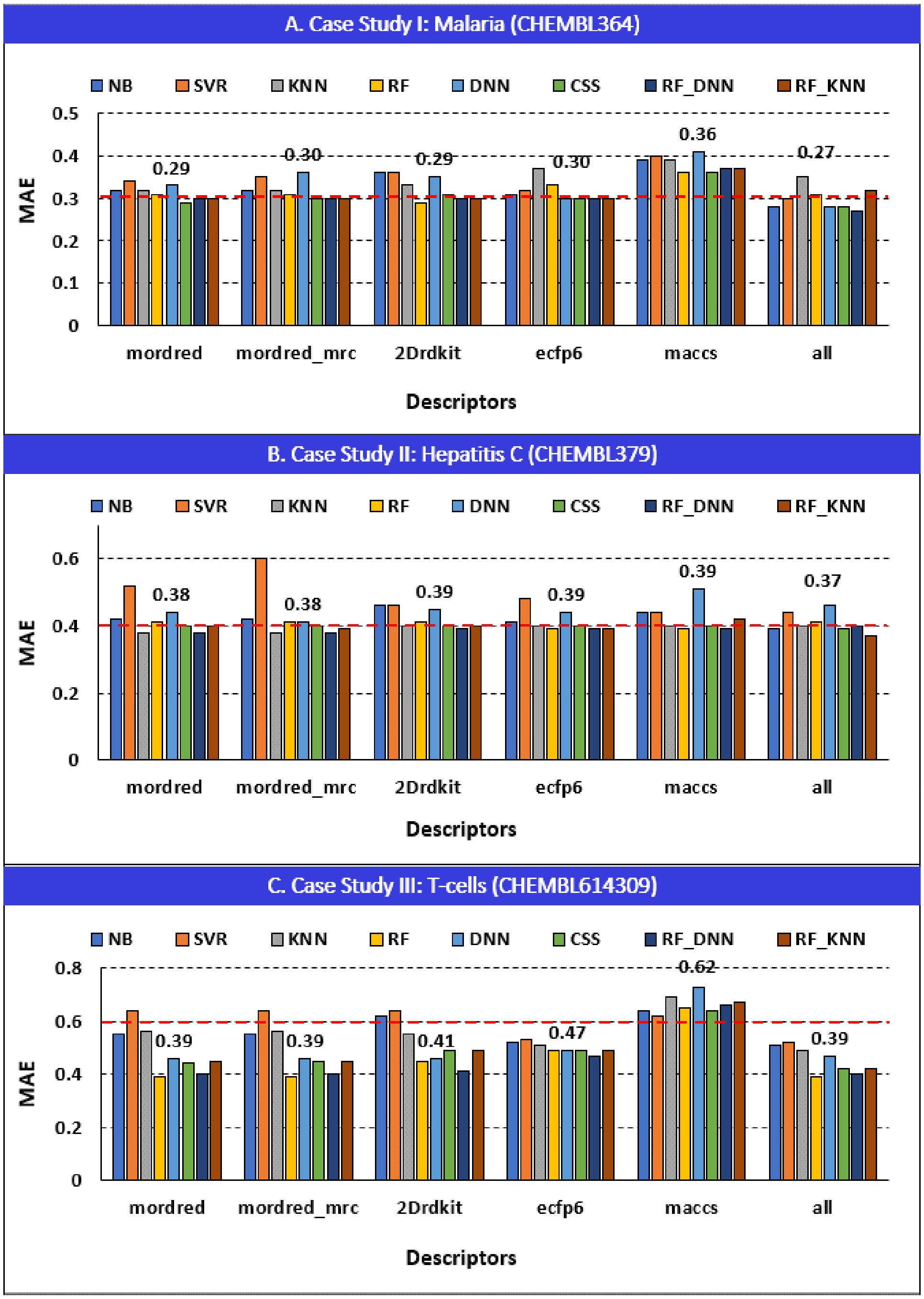

Figure S17. Clustered columns analysis of mean absolute error (MAE) from 10-fold cross validation for (A) Plasmodium falciparum (CHEMBL364), (B) Hepatitis C Virus (CHEMBL379), and (C) T-cells (CHEMBL614309). The numbers indicated on each set of clustered columns are the lowest MAE value from the associated set of 8 machine learning algorithms.

A

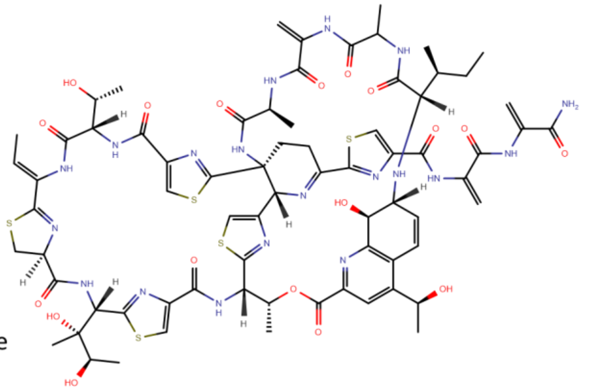

CHEMBL2303629

pIC50 (experimental) = 6.22

27-membered macrolactone

Prediction	NB	SVR	KNN	RF	DNN	CSS	RF_ DNN	RF_ KNN
**mordred**	6.36	5.46	6.53	**6.14**	5.37	5.97	6.33	5.75
**mordred_mrc**	**6.26**	5.28	6.53	**6.17**	6.80	**6.21**	6.35	6.48
**2Drdkit**	8.08	7.72	6.90	**6.26**	8.12	7.42	6.58	7.19
**ecfp6**	5.98	6.05	5.56	6.07	6.05	5.94	5.81	6.06
**maccs**	**6.22**	**6.17**	5.83	6.02	6.08	6.06	5.92	6.05
**all**	7.06	7.32	6.61	**6.15**	6.72	6.77	6.44	6.38

B

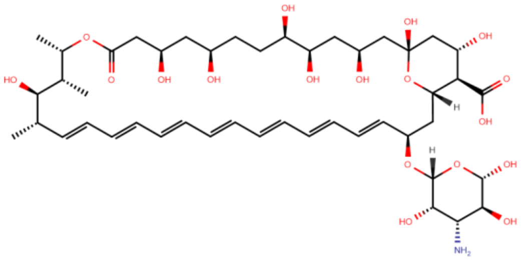

CHEMBL487366

pIC50 (experimental) = 6.1

36-membered macrolactone

Prediction	NB	SVR	KNN	RF	DNN	CSS	RF_ DNN	RF_ KNN
**mordred**	7.41	7.35	6.79	**6.16**	6.68	6.88	6.47	6.42
**mordred_mrc**	7.49	7.82	6.79	**6.17**	7.27	7.11	6.48	6.72
**2Drdkit**	6.25	6.33	6.36	6.34	6.23	6.30	6.35	6.29
**ecfp6**	6.23	6.28	5.67	**6.05**	**6.08**	**6.06**	5.86	**6.06**
**maccs**	5.17	**6.11**	6.52	6.28	6.77	**6.17**	6.40	6.53
**all**	6.41	**6.18**	5.97	**6.12**	6.40	6.21	6.26	**6.04**

Figure S18. Machine Learning workflow predictions on (A) the only 27-membered (CHEMBL2303629) and (B) the only 36-membered (CHEMBL487366) macrolactones from Plasmodium falciparum – malaria (CHEMBL364) ligand dataset. The predicted values within 0.1 of the experimental pIC50 values of the associated macrolactone are bolded.

A

CHEMBL591604

pIC50 (experimental) = 8.08

21-membered macrolactone

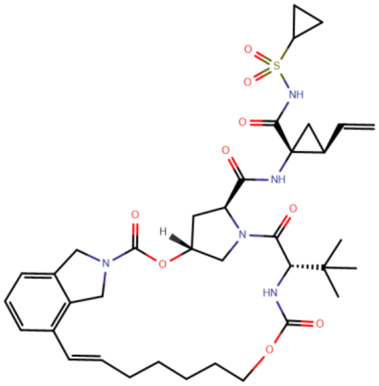

Prediction	NB	SVR	KNN	RF	DNN	CSS	RF_ DNN	RF_ KNN
**mordred**	7.54	7.62	7.85	7.70	7.69	7.68	7.69	7.78
**mordred_mrc**	7.55	7.46	7.85	7.69	7.83	7.68	7.76	7.77
**2Drdkit**	7.48	7.63	7.86	7.83	7.81	7.72	7.82	7.85
**ecfp6**	7.57	7.30	7.13	7.46	7.42	7.38	7.44	7.30
**maccs**	7.57	7.74	7.75	7.64	7.75	7.69	7.70	7.70
**all**	7.60	7.63	**8.01**	7.69	7.48	7.68	7.85	7.58

B

CHEMBL2407590

pEC50 = 7.43

33-membered macrolactone

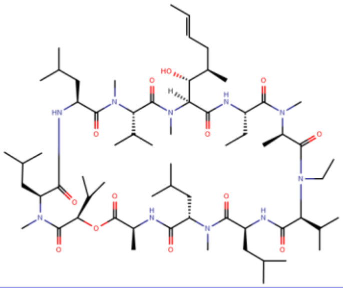

Prediction	NB	SVR	KNN	RF	DNN	CSS	RF_ DNN	RF_ KNN
**mordred**	7.03	8.10	**7.53**	6.46	8.19	**7.46**	7.32	7.00
**mordred_mrc**	7.06	7.60	**7.53**	6.46	6.86	7.10	6.66	6.99
**2Drdkit**	**7.33**	**7.36**	7.53	6.80	4.30	6.67	5.55	7.17
**ecfp6**	5.93	5.37	5.41	6.50	5.36	5.71	5.93	5.95
**maccs**	6.40	5.77	6.17	6.51	4.90	5.95	5.71	6.34
**all**	6.67	5.05	7.92	6.78	5.52	6.39	**7.35**	6.15

Figure S19. 10-fold CV predictions on the (A) 21-membered (CHEMBL591604) and (B) 33-membered (CHEMBL2407590) macrolactones from Hepatitis C Virus (CHEMBL379) macrolactone dataset. The predicted values within 0.1 of the experimental pIC50 values of the associated macrolactone are bolded.

A

CHEMBL162354

pIC50 = 5.37

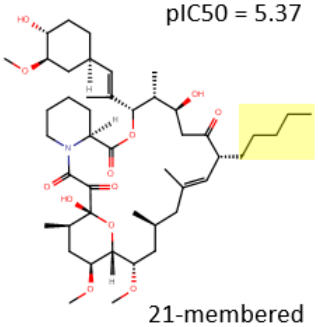

21-membered

R^2	NB	SVR	KNN	RF	DNN	CSS	RF_DNN	RF_KNN
**mordred**	7.25	7.26	7.17	7.04	8.43	7.43	7.10	7.73
**mordred_mrc**	7.25	7.27	7.17	7.04	6.43	7.03	7.11	6.74
**2Drdkit**	7.61	8.22	7.12	7.03	6.24	7.25	7.08	6.64
**ecfp6**	8.31	8.44	8.69	8.60	8.80	8.57	8.65	8.70
**maccs**	7.94	8.24	7.75	7.92	8.32	8.03	7.84	8.12
**all**	8.01	8.15	7.99	7.20	8.28	7.93	7.60	7.74

B

CHEMBL352172

pIC50 = 8.58

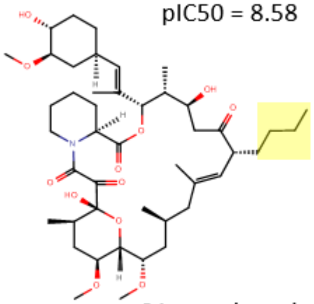

21-membered

R^2	NB	SVR	KNN	RF	DNN	CSS	RF_DNN	RF_KNN
**mordred**	7.41	7.30	7.07	8.01	7.86	7.53	7.54	7.94
**mordred_mrc**	7.41	7.25	7.07	8.05	7.90	7.53	7.56	7.97
**2Drdkit**	7.60	8.14	7.23	7.66	7.48	7.62	7.44	7.57
**ecfp6**	7.29	7.11	8.01	7.16	7.00	7.31	7.58	7.08
**maccs**	7.84	8.23	7.60	7.79	8.27	7.95	7.69	8.03
**all**	7.35	7.17	7.61	8.00	8.68	7.76	7.80	8.34

C

CHEMBL347657

pIC50 = 5.78

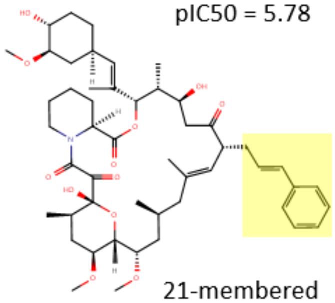

21-membered

R^2	NB	SVR	KNN	RF	DNN	CSS	RF_DNN	RF_KNN
**mordred**	7.14	7.01	8.77	7.57	8.04	7.71	8.17	7.81
**mordred_mrc**	7.14	7.00	8.77	7.59	8.53	7.81	8.18	8.06
**2Drdkit**	8.11	8.59	8.43	8.06	8.39	8.32	8.24	8.22
**ecfp6**	7.11	7.48	6.79	7.27	7.09	7.15	7.03	7.18
**maccs**	8.01	8.27	7.98	7.96	8.24	8.09	7.97	8.10
**all**	7.13	7.50	6.54	7.45	6.45	7.01	7.00	6.95

Figure S20. Machine learning workflow 10-fold CV predictions on the 21-membered macrolactone ligands (A) CHEMBL162354, (B) CHEMBL352172 and (C) CHEMBL347657 from T-cells (CHEMBL614309) dataset. The closest prediction value to the experimental pIC50 of the associated macrolactone are underlined.

Supplemental References

1. Moriwaki, H., Tian, Y.-S., Kawashita, N. & Takagi, T. Mordred: a molecular descriptor calculator. *Journal of Cheminformatics* **10**, 4 (2018).
2. Danielsson, P. E. Euclidean distance mapping. *Computer Graphics and Image Processing* **14**, 227–248 (1980).
3. Szekely, G. J. & Rizzo, M. L. Hierarchical clustering via joint between-within distances: Extending Ward's minimum variance method. *Journal of Classification* **22**, 151–183 (2005).
4. Bastian, M., Heymann, S. & Jacomy, M. Gephi: An Open Source Software for Exploring and Manipulating Networks. *ICWSM* (2009).
5. Jacomy, M., Venturini, T., Heymann, S. & Bastian, M. ForceAtlas2, a Continuous Graph Layout Algorithm for Handy Network Visualization Designed for the Gephi Software. *PLoS ONE* **9**, e98679 (2014).
6. Hu, Y. Efficient and High Quality Force-Directed Graph. *The Mathematica Journal* **10**, 37--71 (2005).
7. Khokhar, D. *Gephi cookbook : over 90 hands-on recipes to master the art of network analysis and visualization with Gephi*.
8. Landrum, G. RDKit Documentation. *Release 2017.09.1* (2017). doi:10.5281/zenodo.60510
9. Onodera, K., Nakamura, H., Oba, Y., Ohizumi, Y. & Ojika, M. Zooxanthellamide Cs: Vasoconstrictive Polyhydroxylated Macrolides with the Largest Lactone Ring Size from a Marine Dinoflagellate of *Symbiodinium* sp. *Journal of the American Chemical Society* **127**, 10406–10411 (2005).
10. Egonw. Class Substructure Fingerprinter. Available at: https://cdk.github.io/cdk/1.5/docs/api/org/openscience/cdk/fingerprint/SubstructureFingerprinter.html.
11. Weissman, J. Polyketide biosynthesis: understanding and exploiting modularity. doi:10.1098/rsta.2004.1470
12. Géron, A. *Hands-On Machine Learning with Scikit-Learn and TensorFlow*. (O'Reilly Media, 2017). doi:10.3389/fninf.2014.00014
13. McKinney, W. & Team, P. D. Pandas - Powerful Python Data Analysis Toolkit. *Pandas - Powerful Python Data Analysis Toolkit* 1625 (2015).
14. van der Walt, S., Colbert, S. C. & Varoquaux, G. The NumPy Array: A Structure for Efficient Numerical Computation. *Computing in Science & Engineering* **13**, 22–30 (2011).
15. Oliphant, T. E., Oliphant, T. E., Oliphant, T. E. & Oliphant, T. Python for Scientific Computing. *Computing in Science & Engineering* **9**, 10–20 (2007).

16. Swain, M. MolVS Documentation. (2018).

17. Mitchell, J. B. O. Machine learning methods in chemoinformatics. *WIREs Comput Mol Sci* **4**, 468–481 (2014).

18. Liaw, A. & Wiener, M. Classification and Regression by randomForest. *R news* **2**, 18–22 (2002).

19. Lowe, R., Glen, R. C. & Mitchell, J. B. O. Predicting phospholipidosis using machine learning. *Molecular Pharmaceutics* **7**, 1708–1714 (2010).

20. Li, Q., Jørgensen, F. S., Oprea, T., Brunak, S. & Taboureau, O. hERG classification model based on a combination of support vector machine method and GRIND descriptors. *Molecular pharmaceutics* **5**, 117–27

21. Doddareddy, M. R., Klaasse, E. C., Shagufta, Ijzerman, A. P. & Bender, A. Prospective validation of a comprehensive in silico hERG model and its applications to commercial compound and drug databases. *ChemMedChem* **5**, 716–29 (2010).

22. Liao, Q., Yao, J. & Yuan, S. Prediction of mutagenic toxicity by combination of Recursive Partitioning and Support Vector Machines. *Molecular diversity* **11**, 59–72 (2007).

23. von Korff, M. & Sander, T. Toxicity-indicating structural patterns. *Journal of chemical information and modeling* **46**, 536–44

24. Sun, H., Shahane, S., Xia, M., Austin, C. P. & Huang, R. Structure based model for the prediction of phospholipidosis induction potential of small molecules. *Journal of Chemical Information and Modeling* **52**, 1798–1805 (2012).

25. Mitchell B.O., J. B. O. Machine learning methods in chemoinformatics. *Wiley Interdisciplinary Reviews: Computational Molecular Science* **4**, 468–481 (2014).

26. Nigsch, F. *et al.* Melting point prediction employing k-nearest neighbor algorithms and genetic parameter optimization. *Journal of Chemical Information and Modeling* **46**, 2412–2422 (2006).

27. Hughes, L. D., Palmer, D. S., Nigsch, F. & Mitchell, J. B. O. Why are some properties more difficult to predict than others? A study of QSPR models of solubility, melting point, and log P. *Journal of Chemical Information and Modeling* **48**, 220–232 (2008).

28. Nemes, L. *et al.* Tailored Similarity Spaces for the Prediction of Physicochemical Properties. *Internet Electronic Journal of Molecular Design* **1**, 150–167 (2006).

29. Kühne, R., Ebert, R. U. & Schüürmann, G. Model selection based on structural similarity - Method description and application to water solubility prediction. in *Journal of Chemical Information and Modeling* **46**, 636–641 (2006).

30. Yap, C. W., Li, Z. R. & Chen, Y. Z. Quantitative structure-pharmacokinetic relationships for drug clearance by using statistical learning methods. *Journal of molecular graphics & modelling* **24**, 383–95 (2006).

31. Basak, S. C. & Grunwald, G. D. Predicting mutagenicity of chemicals using topological and quantum chemical parameters: A similarity based study. *Chemosphere* **31**, 2529–2546 (1995).

32. Kovatcheva, A. *et al.* Combinatorial QSAR of ambergris fragrance compounds. *Journal of Chemical Information and Computer Sciences* **44**, 582–595 (2004).

33. Zakarya, D., Chastrette, M., Tollabi, M. & Fkih-Tetouani, S. Structure-camphor odour relationships using the Generation and Selection of Pertinent Descriptors approach. *Chemometrics and Intelligent Laboratory Systems* **48**, 35–46 (1999).
