# Supplementary figures and images for: Cheminformatics Analysis and Modeling with MacrolactoneDB
Source: Sci Rep. 2020 Apr 14;10:6284. doi: 10.1038/s41598-020-63192-4 (PMC7156526; doi:10.1038/s41598-020-63192-4)

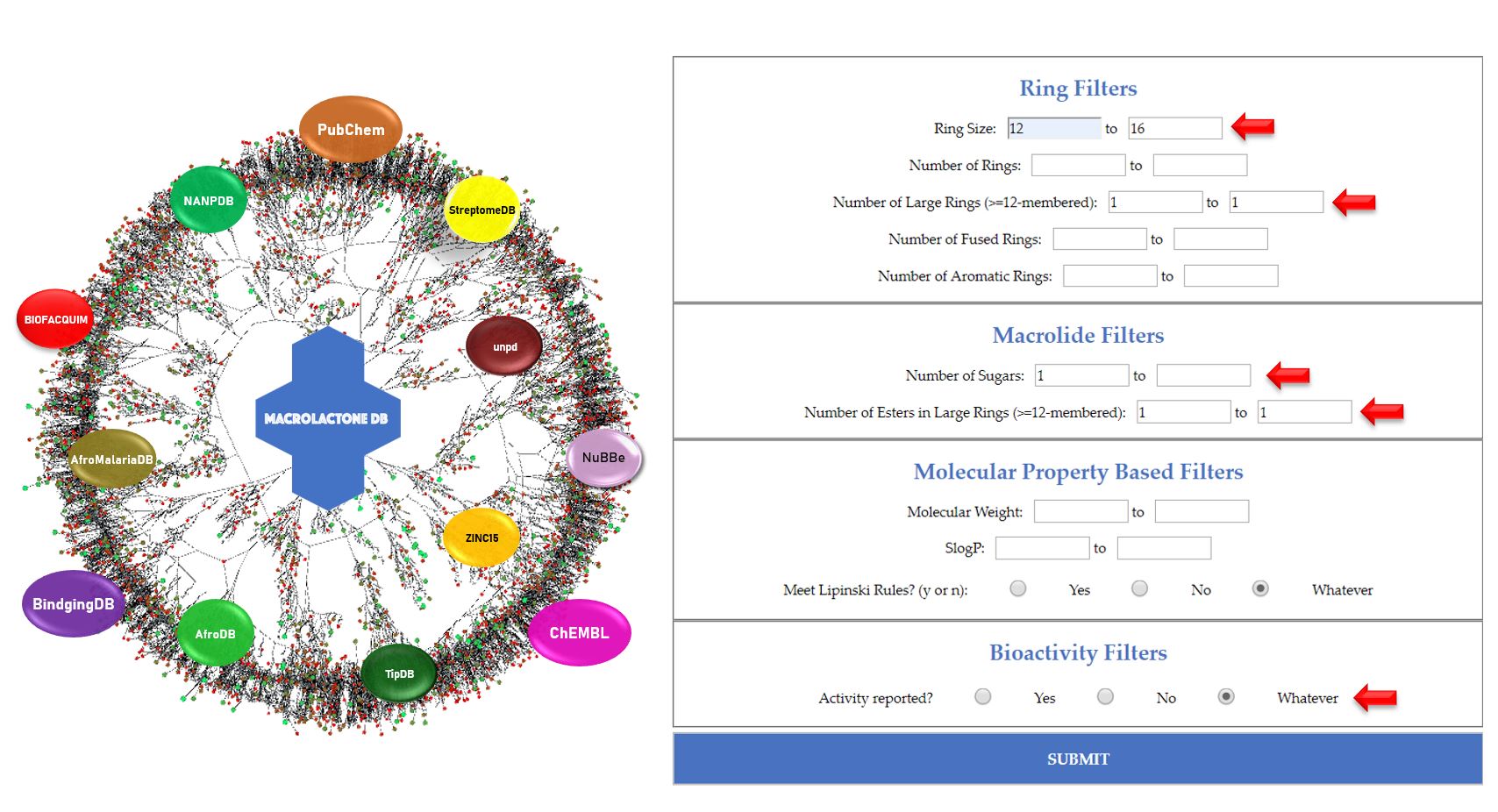

Supplement: Supplementary file 3 — Supplementary information3. [file 41598_2020_63192_MOESM3_ESM.jpg]
